# Hsp70-containing extracellular vesicles are capable of activating of adaptive immunity in models of mouse melanoma and colon carcinoma

**DOI:** 10.1038/s41598-021-00734-4

**Published:** 2021-10-29

**Authors:** Elena Y. Komarova, Roman V. Suezov, Alina D. Nikotina, Nikolay D. Aksenov, Luiza A. Garaeva, Tatiana A. Shtam, Alexander V. Zhakhov, Marina G. Martynova, Olga A. Bystrova, Maria S. Istomina, Alexander M. Ischenko, Boris A. Margulis, Irina V. Guzhova

**Affiliations:** 1 Institute of Cytology of Russian Academy of Sciences, Tikhoretsky Ave. 4, St. Petersburg, Russia 194064; 2 St.Petersburg Nuclear Physics Institute Named By B.P. Konstantinov of National Research Centre «Kurchatov Institute», Orlova roshcha 1, Gatchina, Russia 188300; 3 Institute of Highly Pure Biopreparation of Federal Medical and Biological Agency of Russia, Pudozhskaya street, 7, St. Petersburg, Russia 197110; 4 Federal Almazov North-West Medical Research Centre, Institute of Experimental Medicine, 2 Akkuratova Str., St. Petersburg, Russia 197341

**Keywords:** Cancer microenvironment, Cancer, Cell biology

## Abstract

The release of Hsp70 chaperone from tumor cells is found to trigger the full-scale anti-cancer immune response. Such release and the proper immune reaction can be induced by the delivery of recombinant Hsp70 to a tumor and we sought to explore how the endogenous Hsp70 can be transported to extracellular space leading to the burst of anti-cancer activity. Hsp70 transport mechanisms were studied by analyzing its intracellular tracks with Rab proteins as well as by using specific inhibitors of membrane domains. To study Hsp70 forms released from cells we employed the assay consisting of two affinity chromatography methods. Hsp70 content in culture medium and extracellular vesicles (EVs) was measured with the aid of ELISA. The properties and composition of EVs were assessed using nanoparticle tracking analysis and immunoblotting. The activity of immune cells was studied using an assay of cytotoxic lymphocytes, and for in vivo studies we employed methods of affinity separation of lymphocyte fractions. Analyzing B16 melanoma cells treated with recombinant Hsp70 we found that the chaperone triggered extracellular transport of its endogenous analog in soluble and enclosed in EVs forms; both species efficiently penetrated adjacent cells and this secondary transport was corroborated with the strong increase of Natural Killer (NK) cell toxicity towards melanoma. When B16 and CT-26 colon cancer cells before their injection in animals were treated with Hsp70-enriched EVs, a powerful anti-cancer effect was observed as shown by a two-fold reduction in tumor growth rate and elevation of life span. We found that the immunomodulatory effect was due to the enhancement of the CD8-positive response and anti-tumor cytokine accumulation; supporting this there was no delay in CT-26 tumor growth when Hsp70-enriched EVs were grafted in nude mice. Importantly, pre-treatment of B16 cells with Hsp70-bearing EVs resulted in a decline of arginase-1-positive macrophages, showing no generation of tumor-associated macrophages. In conclusion, Hsp70-containing EVs generated by specifically treated cancer cells give a full-scale and effective pattern of anti-tumor immune responses.

## Introduction

Extracellular vesicles (EVs) serve as a tool for the maintenance of cell/tissue homeostasis in the normal organism or in pathogenesis by the permanent exchange of proteins, lipids, metabolites and microRNA between cells of different origination^1–3^. EVs are comprised of a heterogenous group of particles enveloped by a lipid membrane and according to their origin, may be related to following categories: (a) exosomes, that have a size range between 30 and 120 nm that emerge from the endosomal system, (b) ectosomes or microvesicles with a size range between 80 and 500 nm that are derived from outward budding of the cell plasma membrane and (c) apoptotic bodies that are secreted during the fragmentation of apoptotic cells. Exosomes and microvesicles have an overlapping size range and share common markers (CD63, CD9, CD81) while apoptotic bodies are characterized by an enrichment of phosphatidylserine on their surface^4^.

EVs derived from tumor cells are involved in the promotion of cell proliferation, angiogenesis, metastasis and modulation of the tumor microenvironment^4–6^. EVs were found to inhibit the cytotoxic activity of Natural Killer (NK) cells^7^, lower the proliferation and induce apoptosis of CD8 + cells and promote proliferation and expansion of regulatory T cells^8^.

One of the abundant exosomal and microvesicular markers is a heat shock protein 70 kDa (Hsp70, HSPA1A) that is ubiquitously expressed in cancer cells, most often indicating poor prognosis. It has been proved that the enhanced expression of Hsp70 chaperone leads to the elevation of cell growth rate, escape from programmed cell death, evasion of cell senescence and promotion of metastasis^9^. Tumor protection based on elevated expression of intracellular Hsp70 can be reversed by triggering protein export from cancer cells. The transport of the chaperone to the outer surface of the plasma membrane and extracellular matrix can be initiated by subjecting tumor cells to physical factors such as irradiation, or drug-like chemicals^10–12^. Appearing on the membrane of cancer cells, Hsp70 is targeted by NK cells, whereas Hsp70 persisting in the extracellular matrix or in the blood (in complex with tumor peptides) may penetrate dendritic cells and participate in antigen presentation, ultimately leading to the generation of an adaptive (CD4 + /CD8 +) immune response^13,14^.

We previously reported that extracellular Hsp70 significantly reduced tumor growth and increased the life span of tumor-bearing animals, activating both innate and specific antitumor immunity in two cancer models: mouse melanoma B16 and rat glioma C6 cells^15–18^. This anti-cancer effect was seemingly due to the ability of exogenous Hsp70 (exo-Hsp70) to direct its intracellular analogue to the cell surface and further to the extracellular space. Accumulating evidence suggests that Hsp70 can be secreted from cells within EVs^19^, and this transport appears to be a way to provoke enhanced tumorigenicity; conversely, exosomes filled with Hsp70 possess immunomodulatory activities.

The purpose of the present work was to study how exo-Hsp70 relocates its endogenous analogue to the extracellular space and to which extent the resulting Hsp70-enriched EVs were efficient as immunomodulatory tools.

## Materials and methods

### Hsp70 preparation

Recombinant human Hsp70 was purified from *E. coli* (strain BL21 DE3), transformed with a pMS-Hsp70 plasmid, as previously described^20^. The Hsp70 solution was further detoxified by incubation with Detoxi-Gel Endotoxin Remover Resin (Thermo Scientific, USA) and sterilized by filtration through a 0.22 µm filter (Millipore, USA). According to the E-Toxate assay (Sigma-Aldrich, USA), the level of lipopolysaccharide in the final Hsp70 preparation was lower than 0.25 U/mL. For flow cytometry and confocal experiments, Hsp70 was labeled with Alexa-555 or Alexa-488 or with NHS–biotin (Sigma, USA), according to manufacturer’s instructions. To remove unbound dye (or non-reacted biotin) after labeling the protein was subjected to the dialysis: twice at room temperature for 1 h, and then at 4 °C overnight, stirring.

### Plasmids

Bioimaging was carried out with CT-26_iRFP720_ colon carcinoma cells expressing near far-red fluorescent protein (ex. 698 nm/em. 720 nm) in vivo in Balb/c and in Balb/c nude mice. The pHIV-iRFP720-E2A-Luc was purchased from Addgene, USA Packaging (psPAX2) and envelope (pMD2.G) plasmids were kindly provided by Dr. A. Tomilin (Institute of Cytology of RAS, Russia).

### Cells

Mouse melanoma B16 cells were kindly provided by Prof. L. Sistonen (Turku Centre for Biotechnology, Finland), mouse colon carcinoma CT-26 cells were kindly provided by Prof. G. Multhoff (Technical University of Munchen, Germany) and HEK293FT cells were obtained from the Collection of Cell Lines of the Institute of Cytology RAS, Russia.

B16 and HEK293FT cells were grown in DMEM and CT-26 cells were grown in RPMI-1640 media supplemented with 10% heat inactivated fetal bovine serum (FBS) (HyClone, USA), 2 mM L-glutamine, 100 U/mL penicillin and 0.1 mg/mL streptomycin (PanEco, Russia) in a 5% CO_2_ atmosphere with 90% humidity. Viability was determined by 0.4% trypan blue exclusion.

### Lentivirus production and generation of cell sublines

The HEK293FT cells were co-transfected using PEI (polyethyleneimine, linear, 25 kDa, Polysciences) with a mixture of three plasmids in serum-free DMEM without antibiotics. Virus-containing supernatants were harvested after 24, 48 and 72 h in complete DMEM with 10% FBS, 3 mM Sodium butyrate, 1% non-essential amino-acids (ThermoFisher, USA), 2 mM GlutaMAX (ThermoFisher, USA) and 1% sodium pyruvate (PanEco, Russia), pooled, filtered through a 0.45 µm filter and concentrated using a PEG6000/NaCl solution. The lentivirus titers were determined by counting fluorescently tagged cells according to the protocol at https://www.addgene.org/protocols/fluorescence-titering-assay/. CT-26 cells were transduced with an iRFP720 vector and were cloned in 96-well plates containing feeder of mouse peritoneal macrophages to generate individual single colonies that were analyzed further with aid of VarioSkanLUX (ThermoFischer, USA).

### Analysis of Hsp70 release from B16 cells

**Figure 1:**
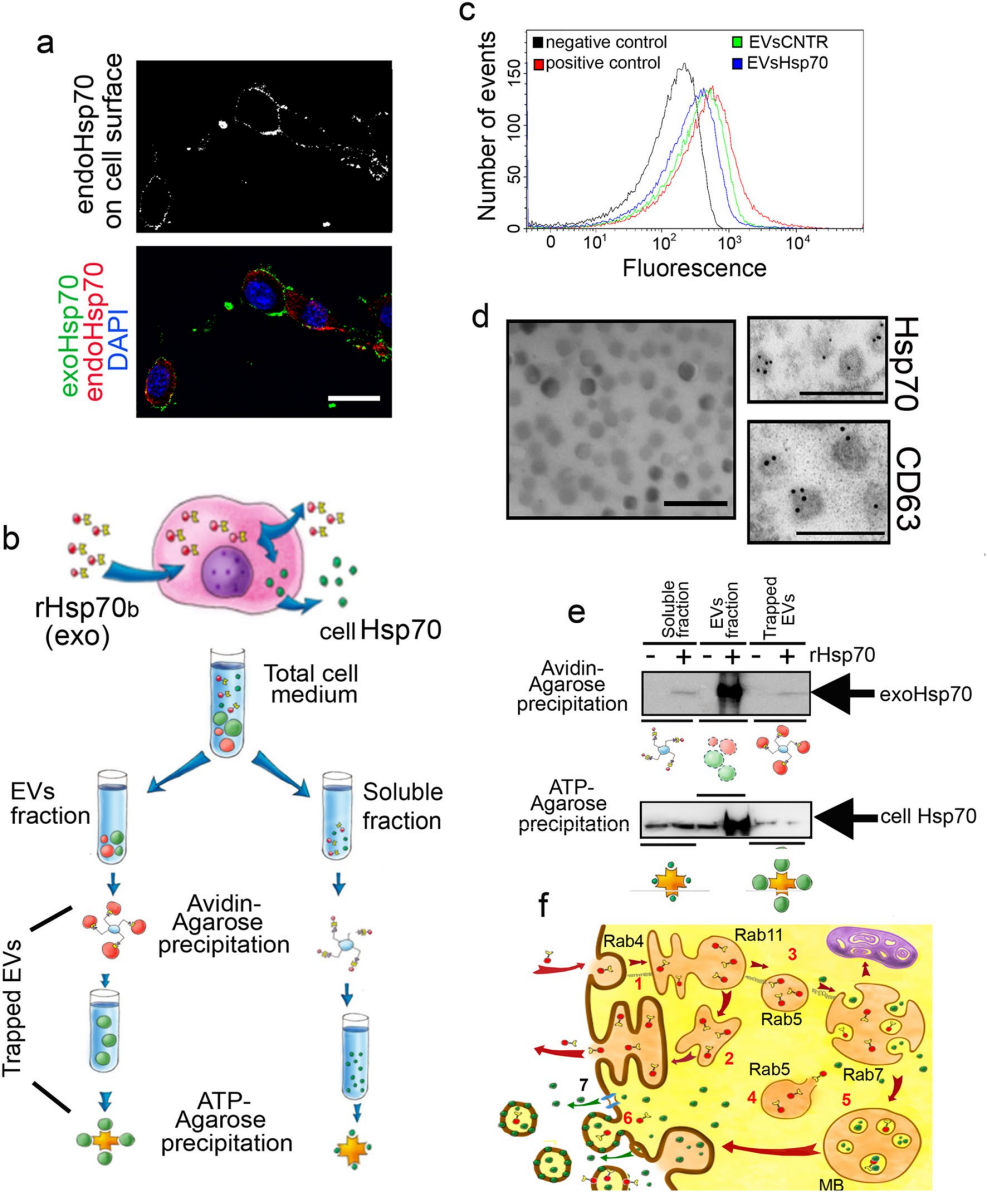
Hsp70 is released from tumor cells in soluble and EV-bound forms. (**a**) B16 cells were incubated with rHsp70-Alexa647 (red) and were then stained with cmHsp70.1 antibody labeled with FITC (green). Nuclei were stained with DAPI (blue). Scale bar: 5 μm; confocal microscopy data. (**d**) Cartoon illustrating the separation of EV-bound and soluble exo- and cell-Hsp70. To discriminate between exogenously introduced Hsp70 and the cellular chaperone, rHsp70 was labeled with biotin and introduced into B16 cell culture for 6 h and then the cells were washed and allowed to export Hsp70 to the extracellular medium for 90 min. Medium was fractionated into EVs and soluble fractions. Both fractions were first incubated with NeutrAvidin-agarose to trap biotinylated Hsp70, and the unbound material was mixed with ATP-agarose to collect the remaining chaperone. The same procedure was employed with the medium of B16 cells without Hsp70 administration. (**b**) EVs were separated as described in Materials and Methods, and analyzed using an Exo-FACS ready-to-use kit for analysis of CD9 positive EVs with flow cytometry. (**c**) EVs were visualized via electron microscopy. For the detection of CD63 and Hsp70 in EVs, TEM microscopy was used with appropriate antibodies. Scale bars: 200 nm. (**e**) Western blotting of separated fractions, as shown in (**d**). NeutrAvidin-precipitated samples were probed with Avidin-peroxidase (upper panel), then ATP-precipitated—with anti-Hsp70 antibody (lower panel). A full-length blot images used to generate the panels are shown in Suppl Fig. S3. (**f**) Paths of exoHsp70 transport inside cells. Upon administration to cell culture, Hsp70 penetrates tumor cells using an endocytosis mechanism and (1) firstly occurs in early Rab4 and Rab11 endosomes, and (2) part of the Hsp70 appears in the extracellular milieu as a result of recirculation. Other Hsp70-containing endosomes mature into Rab5 and Rab7 endosomes (3) and at this stage some stimuli, such as acidification, can cause the release of Hsp70 into the cytoplasm. (4) Occurring in the cytoplasm together with cellular Hsp70, formerly exoHsp70 could be trapped by multivesicular bodies, due to invagination of the membrane of the late endosome (5). Both exo- and cell-Hsp70 in the cytoplasm could be anchored to the plasma membrane and then due to plasma membrane blebbing, could form Hsp70 containing vesicles that cannot be considered as exosomes (6). We cannot exclude the capacity of Hsp70 (exo- or cell-) to be released from cells forming channels (7).

To analyze whether B16 cells could extrude cell-Hsp70 into the extracellular milieu, we used the method described elsewhere^17,18^. Briefly, B16 melanoma cells (1.5 × 10^7^ in each probe) were incubated with 50 µg/mL biotinylated Hsp70, and after 6 h the cells were washed with PBS and placed into serum-free media for the next 90 min. To elucidate which form of exo- and cell-Hsp can be released from B16 cells (i.e. in the soluble fraction or being inside/on the surface of EVs), we collected culture medium which was first centrifuged at 400 g for 5 min to remove dead cells debris, then at 20,000 g for 30 min to remove big vesicles and was finally ultracentrifuged at 110,000 g for 2 h at + 4^o^ C using Ultracentrifuge Beckman Optima XPN-90 (Beckman Coulter Life Sciences, USA) and then Pellet fraction and Supernatant fraction, were used for two-step chromatography. The medium was divided and subjected to a two-step affinity precipitation, first with NeutrAvidin-agarose gel slurry (Pierce) and after the unbound fraction was incubated with ATP-agarose gel (Sigma-Aldrich) (see Fig. 1b and^18^ for details). The gel probes were subjected to treatment with SDS and to western blotting. After electrophoresis in 11% polyacrylamide gel, the protein bands were transferred to a polyvinylidenedifluoride (PVDF) membrane (0.45 μm, Amersham, USA) that was stained alternatively with Avidin-Peroxidase (Pierce, USA) or 3C5 anti-Hsp70 monoclonal antibody (known to recognize human, rat and mouse Hsp70^21^).

### Flow cytometry

To investigate whether exo-Hsp70 in EVs or free exo-Hsp70 collected from the B16 cell medium could re-enter other naïve B16 cells, and could extrude cell-Hsp70 onto cell surface, we incubated naïve B16 cells with Hsp70 labeled with Alexa-647 (as described above) and collected EVs and soluble Hsp70 fractions. The EV pellet was diluted in 0.5 mL of complete medium, whereas the medium containing soluble Hsp70 (10 mL) was concentrated up to final volume of 0.5 mL using the Amicon Ultra-15 Centrifugal Filters Ultracel—30 K (Merck Millipore, Ireland). Media was then transferred to naïve B16 cells and after a 6 h incubation cells were washed and stained with cmHsp70.1-FITC antibody. Analysis was carried out with the aid of a CytoFLEX (Beckman Coulter, USA) flow cytometer using lasers of 488 nm and 647 nm.

Quantitative analysis of the CD9 exosome marker on the surface of the isolated EVs was carried out using an Exo-FACS ready-to-use kit for analysis of exosome markers from cell culture media (Lonza, Estonia) supplied with primary antibodies against CD9 (ab92726, Abcam, UK) and secondary Alexa488-labeled antibodies, according to the manufacturer’s recommendations. The same number of vesicles was added to each sample for flow cytometry, based on the results of measurements of particle concentration using nanoparticle tracking analysis. A sample of PBS, without any EVs, was used as a negative control for non-specific labeling. An aliquot of exosome standard was used as a positive control. Analysis was performed with a CytoFLEX instrument (Beckman Coulter, USA).

### Electron microscopy

To visualize EVs, the EV-containing pellets were resuspended in 1–2 mL of ice-cold PBS and pipetted onto formvar carbon-coated copper single slot grids (Agar Scientific, Stansted, UK) and allowed to adsorb for 20 min. EVs were further fixed with cold 2.5% glutaraldehyde, stained with uranyl acetate, and observed under a Zeiss Libra 120 TEM microscope.

For immuno-electron microscopy (immunogold labeling) the EV-containing pellets were fixed in 2.5% glutaraldehyde in 0.1 M cacodylate buffer, 2% sucrose, for 1.5 h at 4 °C, postfixed in 1% osmium tetroxide in 0.1 M cacodolate buffer (pH 7.4) with 5% sucrose for 1 h at 4 °C, and dehydrated in a graded ethanol series, and embedded in a mixture of Araldite and Epon. Ultrathin sections were cut using an LKB-Ultratome with a diamond knife (Diatome, Ltd., Biel, Switzerland). Sections were mounted on nickel grids and were treated with hydrogen peroxide for 20 min to loosen the resin. Then the sections were incubated with primary antibody overnight, at 4 °C in a moist chamber. Subsequently, the sections were exposed to the secondary antibody for 1 h at room temperature. Finally, the sections were double-stained with uranyl-acetate and lead citrate, and examined by transmission electron microscopy (Zeiss Libra 120, Carl Zeiss, Germany). Control sections were prepared by omitting the primary antibody. The following primary antibodies were used: monoclonal anti-CD63 (diluted 1:200, ab68418, Abcam, UK) and home-made monoclonal anti-Hsp70 (Clone 8D1). The secondary antibody used was gold conjugated (10 nm) anti-mouse IgG (diluted 1:10, Sigma, USA).

### Western blotting

Samples after NeutAvidin-agarose and ATP-agarose chromatography, Hsp70 in EVs and soluble Hsp70 (solHsp70) fractions and B16 cells were analyzed with western blotting. Cells were lysed on ice in RIPA buffer (20 mM Tris–HCl (pH 7.5), 150 mM NaCl, 1 mM EDTA, 0.1% Triton X-100 and 1 mM PMSF). Equal amounts of total protein (20 μg/lane) were electrophoresed with a 10% sodium dodecyl sulfate (SDS) polyacrylamide gel. Proteins were transferred to a polyvinylidene difluoride (PVDF) membrane strips, and non-specific binding on the membrane was blocked with 5% fat-free milk in phosphate-buffered saline (PBS). Membranes were then incubated with anti-Hsp70 monoclonal antibody (Clone 3C5). Glyceral 3-phosphate-dehydrogenase (GAPDH) was used as loading control (ab8245, Abcam, UK). To estimate the level of Hsp70 in EVs, we calculated concentration of EVs using Nanoparticle Tracking analysis EVs and used 8 × 10^9^ EVs for B16 cells and 2 × 10^9^ EVs for CT-26 for western blotting. To analyze the content of arginase-1 in B16 tumors, tumor samples were lysed in RIPA lysing buffer and following electrophoresis and protein transfer to PVDF membranes the membranes were incubated with an antibody to arginase-1 (AF5868, R&D Systems, USA). Chemiluminescent detection of the protein bands was performed with Clarity Western ECL Blotting Substrate and ChemiDoc MP equipment (both Bio-Rad, USA).

### Hsp70-ELISA

Hsp70-ELISA was employed to assess the Hsp70 concentration during biological sample evaluation. *Sample preparation:* The samples for further analysis were prepared as follows: (1) B16 cells (untreated and incubated with rHsp70), as well as CT-26, and CT-26 cells incubated with rHsp70 for 6 h, were washed with cold PBS three times and fresh serum-free medium was added for the next 90 min. The EV fraction was isolated by a series of sequential centrifugations of the conditioned media as described above. To dissolve the EVs for Hsp70 measurement, 100 μl of 0.2 M HCl (pH 3.0) were added to the dry EV pellet following the final centrifugation and the pH of the mixture was immediately altered to pH 7.5 with Tris–HCl (pH 10.0); (2) The supernatant after the last centrifugation, containing Hsp70 in soluble form; (3) B16 cells, untreated and incubated with rHsp70, as well as CT-26 and CT-26 cells incubated with rHsp70 (3.5 × 10^6^ cells/sample) were lysed in RIPA sample buffer, three times freeze-thawed, ultrasonicated and centrifuged (5000 g).

To evaluate Hsp70 content in samples 100 μL of affinity-purified homemade polyclonal anti-Hsp70 antibody (1.5 mg/mL) (see. Suppl Fig. S1) was placed into wells of 96-well plate (Corning, USA) in 20 mM Borate Suppl buffer (pH 8.0), supplemented with 0.15 M NaCl, and incubated for 20 h in a humid chamber. After washing with Buffer A (20 mM Borate buffer (pH 8.0), 0.15 M NaCl, 0.05% Tween-20) the wells were loaded with 100 μL of the appropriate samples as indicated above, and with the rHsp70 titers used for calibration; all probes were mixed with 100 μL of Buffer A. Plates were further incubated for 1 h at 37 °C on a shaker and after washing in Buffer A, biotinylated anti-Hsp70 polyclonal antibody (0.01 μg/mL) was added with an incubation of 1 h at 37 °C on a shaker. After washing StreptAvidin-Peroxidase was added for an additional 1 h. The staining reaction was performed with tetramethylbenzene in citrate buffer (pH 4.5) and the intensity of staining was measured with a Varioscan (Thermo Fisher, USA). All results were obtained in triplicate.

### Cytotoxicity assay

The cytotoxicity assay was performed with the aid of xCELLigence Real-Time Cell Analyzer DC equipment (Hoffmann-La Roche, Switzerland). This impedance-based assay carries out label-free, real-time high-throughput analysis of cell growth and lymphocyte-mediated cytotoxicity^22^. Since NK cells and other lymphocytes are non-adhesive, they do not possess impedance^23^, therefore a change in electrical signal relates only to adherent tumor cells. To evaluate the sensitivity of B16 cells (incubated with Hsp70) to cytotoxic lymphocytes, intact B16 or CT-26 cells were seeded in the wells of an E-plate at concentrations of 10^4^ cells/well and on the next morning rHsp70 (50 μg/mL), or the EVs fraction (concentration of 2.5 × 10^12^ EVs/mL), or soluble Hsp70 extruded from B16 cells (60 ng/mL), were added to intact B16 cells. 6 h later, effector cells isolated from the spleen of C3HA mice were added to the same wells at a ratio of 100:1 (effector:target) and recording was carried out over the next 20 h.

In experiments evaluating the cytotoxic effect of lymphocytes derived from tumor-bearing animals, we washed out cells from the spleens of mice inoculated with B16 cells, with or without EVs and then divided them into two parts: (1) total lymphocyte fraction, (2) lymphocytes that were incubated with DynabeadsFlowComp™ Mouse CD8 (#1146D, Invitrogen, USA) in order to collect CD8 + cells. Cells were washed out from the beads according to manufacturer’s instructions and used as effector cells in the ratio (100:1).

### Animal experiments

All in vivo experimental protocols were approved by the licensing committee of Institute of Cytology of Russian Academy of Sciences**.** (Identification number F18-00,380). All methods were carried out in accordance with relevant guidelines and regulations. All methods are reported in accordance with ARRIVE guidelines.

Female C57BL/6 mice (for subcutaneous B16 melanoma tumor formation), male BALB/c and female BALB/c nude mice (for subcutaneous CT26 tumor formation) were used in this study. C57BL/6 and BALB/c mice were purchased from Biomedical Technology Research Center (Russia), Balb/c nude—from National Research Lobachevsky State University of Nizhny Novgorod (Russia). The C57BL/6 mice were subcutaneously injected with 10^6^ B16 cells, Balb/c—with 2 × 10^5^ CT26_iRFP720_ cells/mouse and /Balb/c nude with 5 × 10^5^ cells. The cells were mixed with EVs, harvested from either untreated cells (EVs-CNTR) or from cells previously incubated with 50 μg/mL rHsp70 (EVs-Hsp70). The number of EVs in the inoculum of the tumor cells was equal to 1 × 10^11^/mouse.

Survival curves were established according to the method of Kaplan and Meier and compared using a generalized Wilcoxon’s test. Tumor growth rate was estimated by weighing tumors taken from control and treated animals on day 19 after engrafting; sera and spleens were collected on the same day. Tumors were photographed, weighed and fixed in 4% formalin. Sera were frozen at − 80 °C before analysis while spleens were used immediately. CT-26 _iRFP720_-injected mice were subjected to bioimaging with the use of the IVIS Spectrum imaging system (Perkin-Elmer, UK) on day 19.

To estimate the specific cytotoxic activity, the splenocytes of mice from all experimental groups were used. For the precise analysis of the total or specific CD8 + cell response, we firstly isolated spleen cells from animals belonging to appropriate treatment groups and further divided each into two groups. One group was incubated with Dynabeads FlowComp™ Mouse CD8 (Invitrogen, USA) to isolate CD8 + cells and the other comprised the total lymphocyte fraction. These cell populations were used as effector cells, which were added to B16 or CT-26 cells at a ratio of 100:1. Cell viability was analyzed using the xCELLigence equipment as described above.

### Immunohistochemistry

To reveal the possible infiltration of B16 tumors with pro-tumor M2 macrophages we prepared serial 7 μm frozen section from tumors isolated from B16-bearing mice and probed the slices with an antibody to arginase-1 (AF5868, R&DSystems, USA) followed by anti-sheep antibody (ab6747, Abcam, UK) and then anti-rabbit antibody conjugated with Alexa488 (Invitrogen, USA). Immunofluorescence images were captured with a Leica TCS SP5 confocal system (Leica Microsystems, Heidelberg, Germany).

### Cytokine measurement in mouse sera

IL-10, IFN-γ and TNF-α measurements were performed with the aid of the following kits: Mouse IL-10 ELISA (#88–7105-88, Invitrogen, USA), Mouse IFN-γ ELISA (#88–7314-88, Invitrogen, USA), and Mouse TNF-α ELISA (#88–7324-88, Invitrogen, USA) according to the manufacturer’s instructions. All the probes were triplicated.

### Nanoparticle tracking analysis (NTA)

The size of EVs and their concentration were determined using the NTA NanoSight® LM10 (Malvern Instruments) analyzer, equipped with a blue laser (45 mW at 488 nm) and a C11440-5B camera (Hamamatsu Photonics K.K., Japan). Recording and data analysis were performed using the NTA software 2.3. To optimize the measurement mode, the samples of isolated vesicles were diluted 1:100, 1:1000, or 1:10,000 by PBS. In the selected dilution, each sample was measured in triplicate. The following parameters were evaluated during the analysis of recordings monitored for 60 s: the average hydrodynamic diameter, the mode of distribution, the standard deviation, and the concentration of vesicles in the suspension.

### Statistical analysis

All numerical results are reported as the mean ± standard error of the mean (SEM) and represent data from a minimum of three independent experiments. Quantitative analysis was performed with the use of Graph Pad Prism 8.0 (Graph Pad Software Inc). The one-way ANOVA test followed by Dunnett’s multiple comparison test (which compares the mean of each condition with control) was used. Differences were considered statistically significant at *p* < 0.05.

### Ethic approval and consent to participate

All in vivo experiments were carried out following the requirements of the Institute of Cytology of Russian Academy of Sciences Ethic Committee (Identification number F18-00,380).

## Results

### Hsp70 penetrates B16 cells using endocytosis and is released in soluble and EV-bound forms

Previously, we demonstrated that the Hsp70 chaperone uses distinct mechanisms to penetrate cells; a major way in human neuroblastoma SK-N-SH and rat glioma C6 cells was endocytosis, whereas in human erythroleukemia cells, K562, Hsp70 entered mainly using lipid rafts^18,24^. To investigate which transport mechanism is employed by the exogenous chaperone in B16 cells, we used flow cytometry analysis with endocytosis inhibitors and a fluorochrome-labeled Hsp70 (Suppl Fig. S2). We also used confocal microscopy to visualize B16 cells transiently transfected with endosome marker genes (Suppl Fig. S2), and demonstrated that the preferable way for exoHsp70 to enter B16 cells was by clathrin-dependent endocytosis.

Importantly, incubation with exo-Hsp70 caused a powerful release of its intracellular analogue to the cell surface, as was shown using confocal microscopy (Fig. 1a). In order to understand in which form both endo-Hsp70 or cell-Hsp70 and exo-Hsp70 are released from the tumor cell, we carried out chromatographic separation of exo- and cell-Hsp70, a method utilised in our previous studies^17,18^, with modifications (Fig. 1b). Suggesting that a part of releasing Hsp70 molecules may be embedded into EVs we subjected the conditioned medium of B16 cells (untreated or incubated with biotinylated recombinant Hsp70 (rHsp70) to centrifugation at the speed necessary for EVs sedimentation. Two samples of vesicles isolated from conditioned culture medium of B16 cells (untreated and incubated with rHsp70) were analyzed by NTA to estimate their size and concentration. The mode size of the vesicles isolated from both conditioned culture medium of B16 cells was in a range from 77 to 97 nm, which corresponded well with the size of exosomes^25^. The concentration of vesicles in the final ultracentrifuged samples was amounted to 2–4 × 10^12^ particles/mL (Suppl Table S1). The presence of CD9 exosome marker on the surface of the vesicles was estimated in parallel in the samples of vesicles isolated from both B16 cells using the same exosome standard as a positive control and beads non-incubated with any vesicles as a negative control (Fig. 1b). To visualize the EVs we used electron microscopy and immuno-electron microscopy (Fig. 1c). According to electron microscopy data EVs were CD63 and Hsp70 positive (Fig. 1d).

Both fractions (i.e. the pellet containing the EVs and the supernatant containing soluble forms of the chaperone) were first incubated with NeutrAvidine-Agarose to trap former exo-Hsp70, or exo-Hsp70 which was located on the EV surface. The unbound fraction was loaded onto ATP-Agarose gel to bind any remaining chaperone. The fractions, together with unresolved EVs, were subjected to immunoblotting using Streptavidin-peroxidase to reveal former exoHsp70 (biotinylated), and the ATP-Agarose-bound proteins were probed with 3C5 (known to recognize all forms of Hsp70)^21^. The biotinylated (exo)-Hsp70 was found in the soluble fraction, inside EVs and even on the EV surface, causing some of the EVs to be trapped within the NeutrAvidin-Agarose (Fig. 1e). ATP-agarose also trapped Hsp70 following NeutrAvidin-Agarose precipitation in all samples, and from this data we suggest that both exo- and cell-Hsp70 leave tumor cells in both soluble- and EV-bound forms. Moreover, chaperones of both kind of origin were found on the EV surface, since intact EVs were trapped with both Agaroses (Fig. 1e). These results are schematically represented in Fig. 1f. Importantly, soluble Hsp70 fraction was not contaminated with EVs as well as EVs fraction did not contein soluble chaperone (Suppl Fig. S4).

### Soluble and EV-bound Hsp70 sensitize B16 cells to NK cells

**Figure 2:**
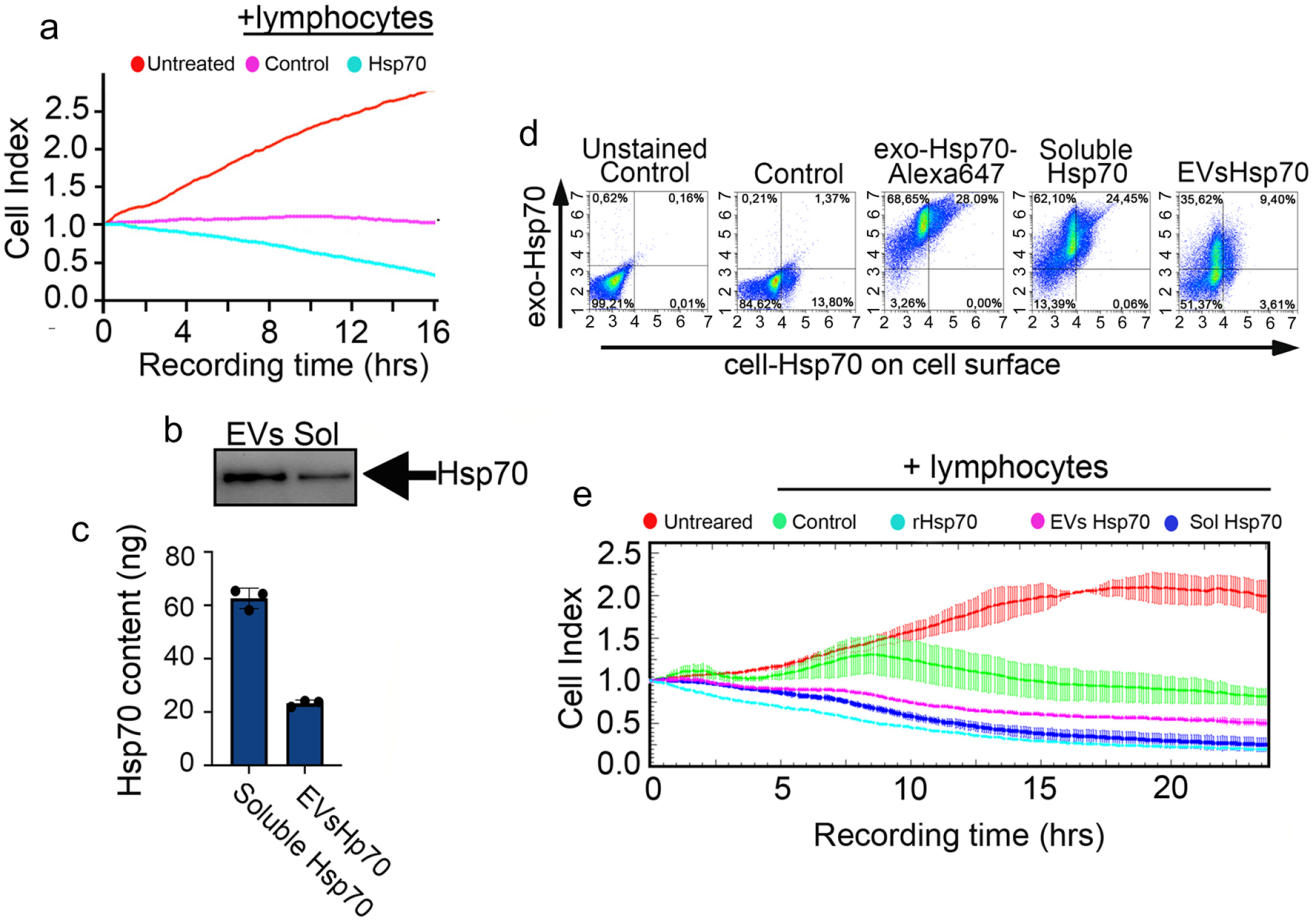
Both soluble Hsp70 and EVs Hsp70 pull out intracellular (endo) Hsp70 to the cell surface and sensitize B16 cells to cytotoxic lymphocytes. (**a**) B16 cells were incubated with rHsp70 for 6 h and were subjected to the CTL test with lymphocytes from a healthy mouse. Cell death was recorded in real time with the aid of xCELLigence. (**b**) EVs and soluble fractions from B16 cells incubated with rHsp70 were collected and subjected to (**b**) Western blotting or (**c**) Hsp70-ELISA. Data from three independent experiments are presented. A full-length blot images used to generate the panel is shown in Suppl Fig. S5 (**d**) B16 cells were incubated with rHsp70-Alexa647, EVs Hsp70 and soluble Hsp70, and subjected to flow cytometry. Representative data of three independent experiments is presented. (**e**) B16 cells were incubated with the soluble fraction or with EVs-Hsp70 for 3 h and lymphocytes of an untreated mouse were added. rHsp70 (50 μg/mL) was used as a positive control. The CTL assay was employed with the aid of xCELLigence. Representative data of three independent experiments is presented.

Hsp70 exported onto the surface of tumor cells (Fig. 1a) was previously shown to activate the cytotoxic response of NK cells^26^. We confirmed this in experiments where B16 cells were incubated with the extracellular chaperone and were then subjected to attack of lymphocytes which were isolated from C3HA mice (Fig. 2a). A Cytotoxic T lymphocyte (CTL) assay was performed with the use of the xCELLigence technique, which allowed us to trace the cell populations’ behaviour in real time, and revealed that exoHsp70 reduced the quantity of live B16 cells which were exposed to cytotoxic lymphocytes. Importantly, lymphocytes did not cause any changes in Cell Index (data not shown)^22^, so a signal change related only to adherent tumor cells, whose characteristics were measured in this test.

To check which form of extracellular Hsp70 can penetrate naïve B16 cells and cause the immune cell reaction we first incubated the naïve B16 with Alexa647-labelled Hsp70 and the conditioned medium was separated in soluble and EV fractions, as described above. Using western blotting (Fig. 2b) and Hsp70-ELISA (Fig. 2c), we measured the Hsp70 content in the two fractions and found that the amount of Hsp70 in the EV-fraction was 25.5 ± 0.5 ng/mL, whereas in the soluble fraction it was more than two-fold higher; 59.6 ± 2.9 ng/mL.

Both fractions were introduced to fresh (naïve) B16 cells which were then stained with a cmHsp70.1 antibody exclusively recognizing Hsp70 located on the surface of cancer cells, and following this cells were subjected to flow cytometry analysis. We found that even in non-treated B16 cells, Hsp70 was exposed on the surface of approximately 15% of cells. A treatment with 50 μg/mL of rHsp70 led to the appearance of Hsp70-Alexa647 inside 97% of cells and also increased the amount of cells with surface Hsp70 up to 28.1%. Upon administration of soluble Hsp70, we found 93% of cells were Alexa647-positive and 25.5% of the cells carried Hsp70 on the surface. Administration of the EV-fraction to B16 cells led to the appearance of Hsp70-Alexa647 in 45.02% of cells and surface staining was demonstrated by 13% of cells (Fig. 2d).

The sensitivity of B16 cells incubated with soluble Hsp70, EVs-Hsp70 or rHsp70 to CTL was measured with the aid of xCELLigence equipment. During 24 h of recording, the number of untreated B16 cells doubled, whereas B16 cells incubated with CTL had a Cell Index equal to 0.77, which means that the number of tumor cells had decreased by about a quarter by the end of the observation. However, in the first 8 h after the addition of lymphocytes, the number of cells did not differ significantly from cells in the control wells (Fig. 2e). Preliminary incubation with either soluble/EVs-bound Hsp70, or with 50 μg/mL rHsp70, led to a decrease in the B16 cell population up to a Cell Index equal to 0.25 for B16 cells incubated with soluble Hsp70 and rHsp70, and up to 0.505 for EVs-Hsp70 (Fig. 2e). The data from these experiments made us to suggest that both soluble Hsp70 and EVs-Hsp70 demonstrate penetrating and immunomodulatory activities close to that of rHsp70, despite being of a 1000-fold lesser concentration. Interestingly, when we added soluble Hsp70 and EVs-Hsp70 in equal amount to B16 cells EVs sensitized B16 cells to NK cells more effectively that soluble Hsp70 (Suppl Fig. S6).

Thus, Hsp70 released from cells stimulated by such strong extrusive factor, as the exogenous chaperone, occurs in intracellular space in two forms: soluble and associated with EVs. In view of the contradictory modern literature regarding the link between Hsp70 and EVs in tumor progression, in this work we focused on the analysis of the properties of this particular protein fraction.

### Administration of EV-Hsp70 does not affect B16 cell proliferation but delays tumor growth in vivo

**Figure 3:**
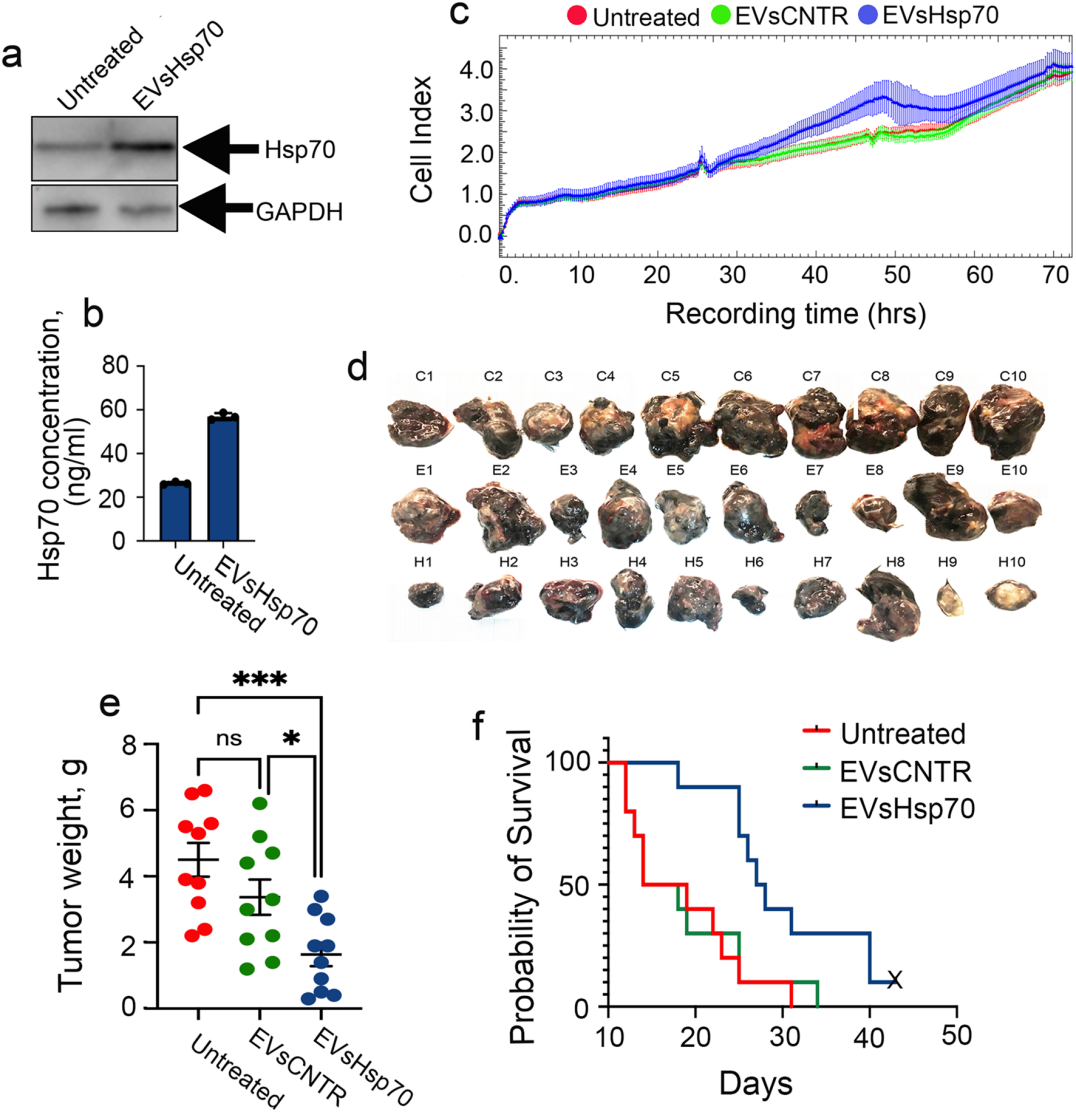
Hsp70-containing EVs do not affect B16 cell growth in vitro, but inhibit growth and increase survival rate of B16-bearing mice*.* The Hsp70 content in B16 cells, untreated and incubated with rHsp70 measured using (**a**) western blotting and (**b**) with a Hsp70-ELISA assay. A full-length blot images used to generate the panels on b are shown in Suppl Fig. S5. (**c**) EVs-CNTR or EVs-Hsp70 were added to B16 cells and their proliferation was measured using xCELLigence. B16 melanoma cells were used to inoculate C57Bl/6 mice, together with EVs-CNTR or EVs-Hsp70, after 19 days tumors were isolated, photographed (**d**) and weighed (**e**). ***p = 0.0006; *p = 0.0404, Dunnett’s multiple comparison test. (**f**) Cumulative proportion Kaplan–Meier curve. Survival was analyzed in the ’Untreated’ group (n = 10), ‘EVs-CNTR’ group (n = 10) and ‘EVs-Hsp70’ group (n = 10).

The exosomes containing Hsp70 are thought to play controversial roles in tumor growth and therefore in this study we explored the effects of Hsp70-containing EVs released from B16 cells treated with the chaperone. First, using western blotting and Hsp70-ELISA we proved, that vesicles could introduce additional amounts of the chaperone to tumor cells (Fig. 3a, b). However, the elevation of the Hsp70 content in cells did not result in an additional growth capacity. The proliferation assay performed using xCELLigence demonstrated that proliferation of B16 with an increased content of Hsp70 was not elevated significantly, compared with untreated cells or those incubated with EV-CNTR B16 cells (Fig. 3c).

Next we analyzed the effect of Hsp70-containing EVs on the progression of B16 melanoma cells in vivo. We collected 2 × 10^11^ EVs from untreated B16 cells and from B16 cells preincubated with rHsp70, and mixed each portion of the EVs with 2 × 10^7^ intact B16 cells. The C57/6 black mice in each group were inoculated subcutaneously with 10^6^ cells/animal. There were three experimental groups with 20 mice in each: mice with untreated B16 cells, mice injected with B16 cells in presence of ‘EVs-CNTR’ and mice with B16 cells in presence of ‘EVs-Hsp70’. The mice did not have any other treatment in the next 19 days, during which, 10 animals from each group were randomly selected to measure tumor weight and to evaluate their immunological characteristics. The other 10 animals from each group were left for survival evaluation.

The tumors collected on day 19 had different sizes and different weights (Fig. 3d,e); the average weight of tumors was 4.5 ± 0.5 g from the untreated group, 3.4 ± 0.5 g from tumors inoculated with EVs-CNTR, and 1.6 ± 1.4 g from tumors inoculated with EVs-Hsp70, differing significantly (*p* < 0.001) from the untreated group.

The delay in tumor growth in the ‘EVs-Hsp70’-treated group resulted in prolonged survival. The death of mice belonging to the ‘Untreated’ and ‘EVs-CNTR’-treated groups occurred within 17 to 36 days (average of 23.6 ± 2.1 days) and 17 to 39 days (average of 23.8 ± 2.4 days), respectively, whereas in the ‘EVs-Hsp70’-treated group, the death outcome was postponed to 23 to 45 days (average of 33.7 ± 2.5 days) for 9 animals and one mouse from this group lived for three additional months (Fig. 3f).

**Figure 4:**
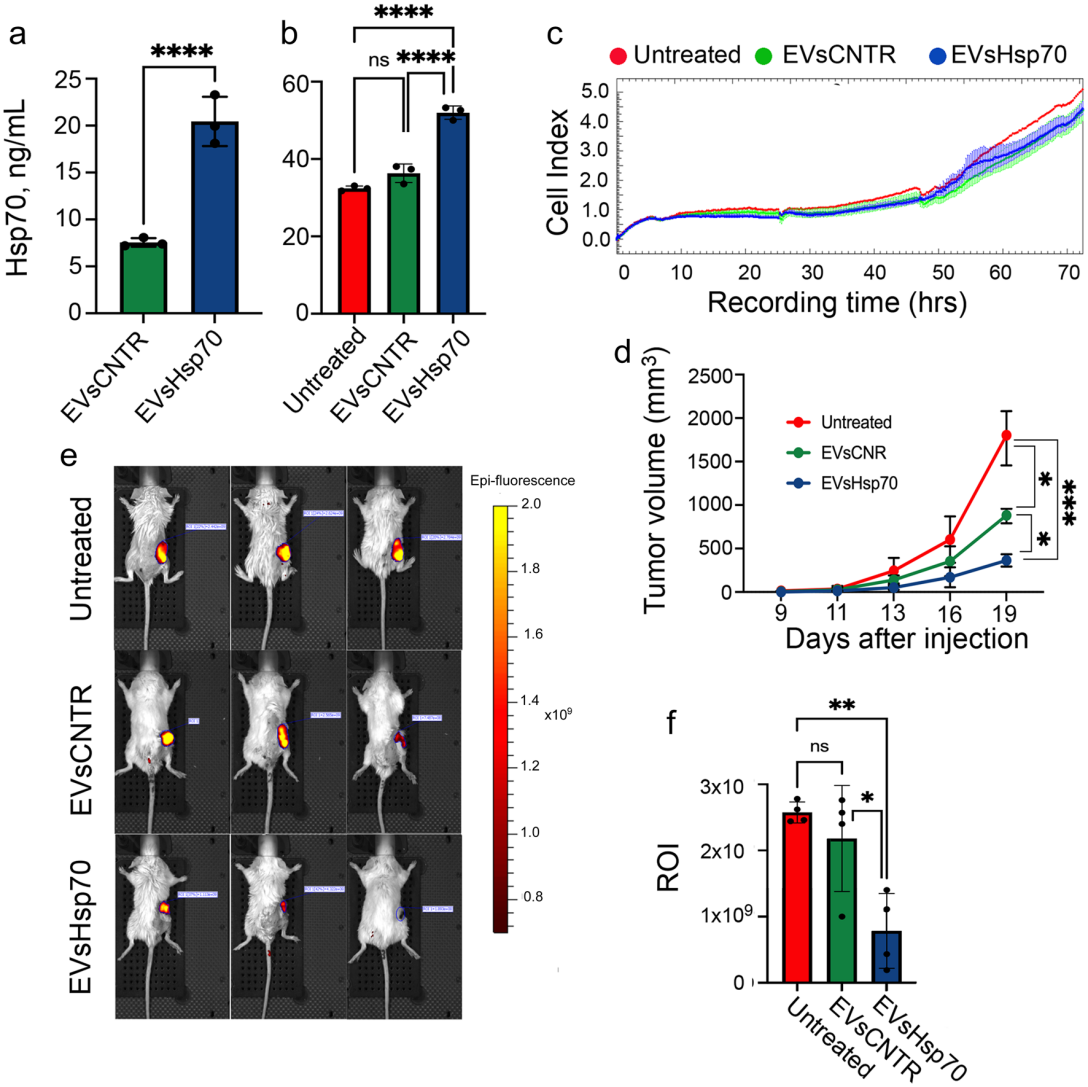
The antitumor effect of EVs from CT-26 cells depends on the Hsp70 content. (**a**) The level of Hsp70 was measured in EVs or (**b**) in cell lysates from CT-26, untreated and incubated with rHsp70, with the aid of Hsp70-ELISA. ****p < 0.0001. (**c**) EVs-CNTR or EVs-Hsp70 were added to CT-26 cells and their proliferation was measured using xCELLigence. (**d**) Tumor volume was measured each 2nd day, starting from day 9 in Balb/c mice (n = 10 in each group) ***p = 0.0003; *p = 0.0371; 0.0428. (**e**) Tumors were monitored using the IVIS Spectrum imaging system (filters: excitation: 675 nm, emission: 720 nm). Representative biofluorescence images. (**f**) Fluorescence count of tumor lesions, (**p = 0.0043, *p = 0.0182, Dunnett’s multiple comparison test).

Melanoma is a tumor that responds well to immunotherapy with Hsp70^27^. To understand whether the effect of tumor growth retardation in the presence of EVs-Hsp70 would be repeated in other types of malignant neoplasms, we used mouse colon carcinoma CT-26 cells. As expected, the treatment with rHsp70 led to a significant elevation of Hsp70 in EVs (20.4 ± 1.5 vs 7.6 ± 0.3 ng/mL) (Fig. 4a) that was confirmed with western blotting (Suppl Fig. S8).

We collected the EVs from CT-26, and CT-26 cells incubated with rHsp70, (characterized in Suppl Table S1, and Suppl Fig. S9), and measured Hsp70 content in EVs-CNTR and EVs-Hsp70 using Hsp70 ELISA (Fig. 4a). Hsp70 amount in EVs-Hsp70 was significantly higher than in EVs-CNTR (20.44 ± 1.51 vs 7.65 ± 0.27 ng/mL). This result was confirmed with western blotting data (Suppl Fig. S8 Incubation of CT-26 cells with 10^12^ EVs of each type led to Hsp70 elevation of intracellular chaperone; Hsp70 content in cells incubated with EVs-Hsp70 significantly (*p* < 0.0001) was higher than in ‘Untreated’ CT-26 cells or cells incubated with EVs-CNTR (Fig. 4b). However this fact did not affect the growth characteristics of CT-26 cells, which were sawed in the wells of the E-plate with EVs-CNTR or EVs-Hsp70 (Fig. 4c). But when we subjected EVs to CT-26 cells in a CTL test using xCELLigence we were convinced that as well as in B16 melanoma cells, the sensitivity of CT-26 cells was directly proportional to the quantity of Hsp70 in EVs (Suppl Fig. S10).

To check whether Hsp70-bearing EVs may delay tumor growth in vivo we used CT-26 cells transfected with an iRFP720 construct. Mice were randomly divided into three groups, with 10 mice in each group. For inoculation we mixed 1.5 × 10^6^ CT-26_iRFP720_ cells with 0.5 × 10^12^ EVs from CT-26 and from CT-26 cells previously incubated with rHsp70, then inoculated 2 × 10^5^ cells/animal subcutaneously.

Assessing changes in tumor volume each second day, we demonstrated that the tumor growth in the ‘EVs-Hsp70’ group was delayed and by the 18th day after inoculation, was approximately fourfold less than in the ‘Untreated’ group (Fig. 4d). This result was confirmed with the aid of bioimaging, utilizing the IVIS equipment (Fig. 4e) and which was performed on day 19 after inoculation. Compared to the ‘Untreated’ group, mice that received ‘EVs-Hsp70’ demonstrated reduced biofluorescence (0.54 ± 0.07 × 10^9^ vs 2.57 ± 0.16 × 10^9^ fluorescence counts) (Fig. 4f). This data proves that the antitumor activity of EVs is dependent on the Hsp70 content in the vesicles.

### Anticancer effects of Hsp70-containing EVs is due to the generation of a specific immune response

To check whether tumor growth delay in the presence of Hsp70-bearing EVs was associated with antitumor immunity, we isolated lymphocytes from the spleens of tumor-bearing animals (on day 19) and divided the lymphocyte populations into two parts. The first part represented the total fraction of lymphocytes, while the second part was magnetically separated and contained CD8 + cells.

**Figure 5:**
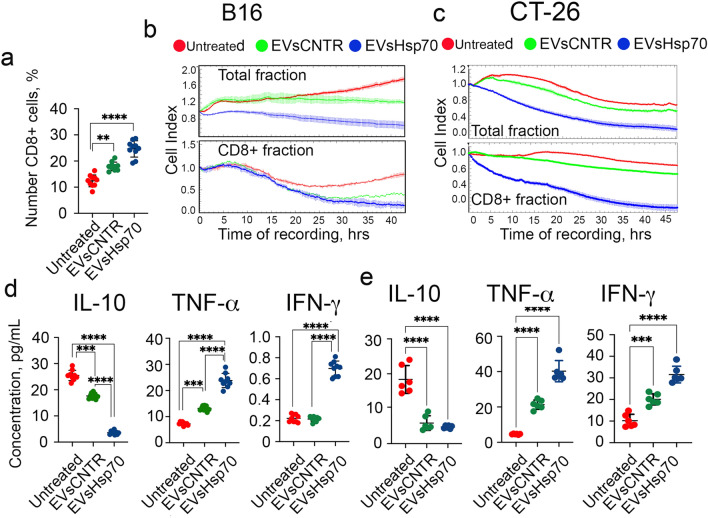
Hsp70-containing EVs cause the generation of a specific immune response. (**a**) Lymphocytes from B16 tumor-bearing mice were isolated from spleens, magnetically separated and the CD8 + cells were counted (**p < 0.0001, Dunnett’s multiple comparison test). (**b**, **c**) Lymphocytes from B16 tumor-bearing mice from three groups (**b**) and from CT-26-bearing mice (**c**) were used as effector cells in the CTL assay performed with the aid of xCELLigence (total fraction, upper panel) and from the CD8 + lymphocyte fraction (lower panel). (**d**, **e**) The concentration of three cytokines (IL-10, TNF-α and IFN-γ) were measured in sera of B16-bearing mice (**d**) and CT-26-bearing mice (**e**) on day 19 after tumor cell inoculation. (*p = 0.0003; **p < 0.0001, Dunnett’s multiple comparison test).

First, we noticed that the share of CD8 + cells in the total splenocyte population in mice bearing B16 tumors was higher in animals inoculated with tumor cells in the presence of EVs. In mice with ‘EVs-CNTR’ the proportion of CD8 + lymphocytes was 17.9 ± 0.5%, whereas in untreated animals it was 12.6 ± 0.8% (*p* < 0.001) and it was even higher in mice obtained EVs-Hsp70 (24.6 ± 1.0%) (Fig. 5a).

Lymphocytes from two fractions were used as effector cells in the CTL assay with intact B16 cells and the CTL effect was recorded using xCELLigence (Fig. 5b). We found that when lymphocytes from the total fraction were used as effector cells, the highest antitumor activity was shown for lymphocytes from animals of the ‘EVs-Hsp70’group. Interestingly, the cytotoxic activity of lymphocytes from the ‘EVs-CNTR’ group did not differ from that of the ‘Untreated’ group within a period of the first 20 h of recording, however, cell death became significantly different in both populations (Fig. 5b, upper panel).

We observed a similar effect when selected CD8 + lymphocytes were used as effector cells. The toxic effect of these cells was higher, even in the ‘Untreated’ cell population, when compared with the CTL of the total fraction. CD8 + lymphocytes from the ‘EVs-Hsp70’ group decreased the Cell Index of target B16 cells down to 0.205, differing significantly from the ‘Untreated’ group (Fig. 5b, low panel).

The effect of Hsp70 content on EVs activity was more pronounced in experiments using lymphocytes isolated from CT-26-bearing mice (Fig. 5c). Data from xCELLigence showed that lymphocytes from animals of ‘EVs-Hsp70’ group demonstrated the highest cytotoxic activity if to compare with that of the ‘Untreated’ and ‘EVs-CNTR’ group, although cytotoxic activity of lymphocytes from ‘EVs-CNTR’ group also was significantly higher than in ‘Untreated’ group. A very similar pattern of cytotoxicity (induced by EVs) was observed when CD8 + cells were employed as effector cells.

Suggesting that the activation of Hsp70-containing EVs was due to increased cytokine production, we measured IL-10, TNF-α and IFN-γ concentrations in serum samples taken from B16 and CT-26 mice injected with EVs (Fig. 5d,e). As expected, the pro-tumor IL-10 level was significantly lower in the serum of animals from the ‘EVs-Hsp70’ group than in serum of mice from the ‘Untreated’ groups (3.6 ± 0.2 vs 25.5 ± 0.6 pg/mL, *p* = 0.000001 for B16 and 4.8 ± 0.2 vs 18.4 ± 1.6 pg/mL for CT-26, *p* = 0.0001). TNF-α and IFN-γ demonstrated an opposite tendency: the level of both cytokines was elevated in the serum of the ‘EVs-Hsp70’ group compared with the “Untreated’ group (23.8 ± 0.9 vs 7.2 ± 0.2 pg/mL in B16 mice and 40.3 ± 2.4 vs 4.6 ± 0.1 pg/mL in CT-26 mice for TNF-α, and 0.2 ± 0.01 vs 0.7 ± 0.02 pg/mL in B16 mice and 31.6 ± 1.6 vs 10.2 pg/mL in CT-26 mice for IFN-γ (Fig. 5d, e).

**Figure 6:**
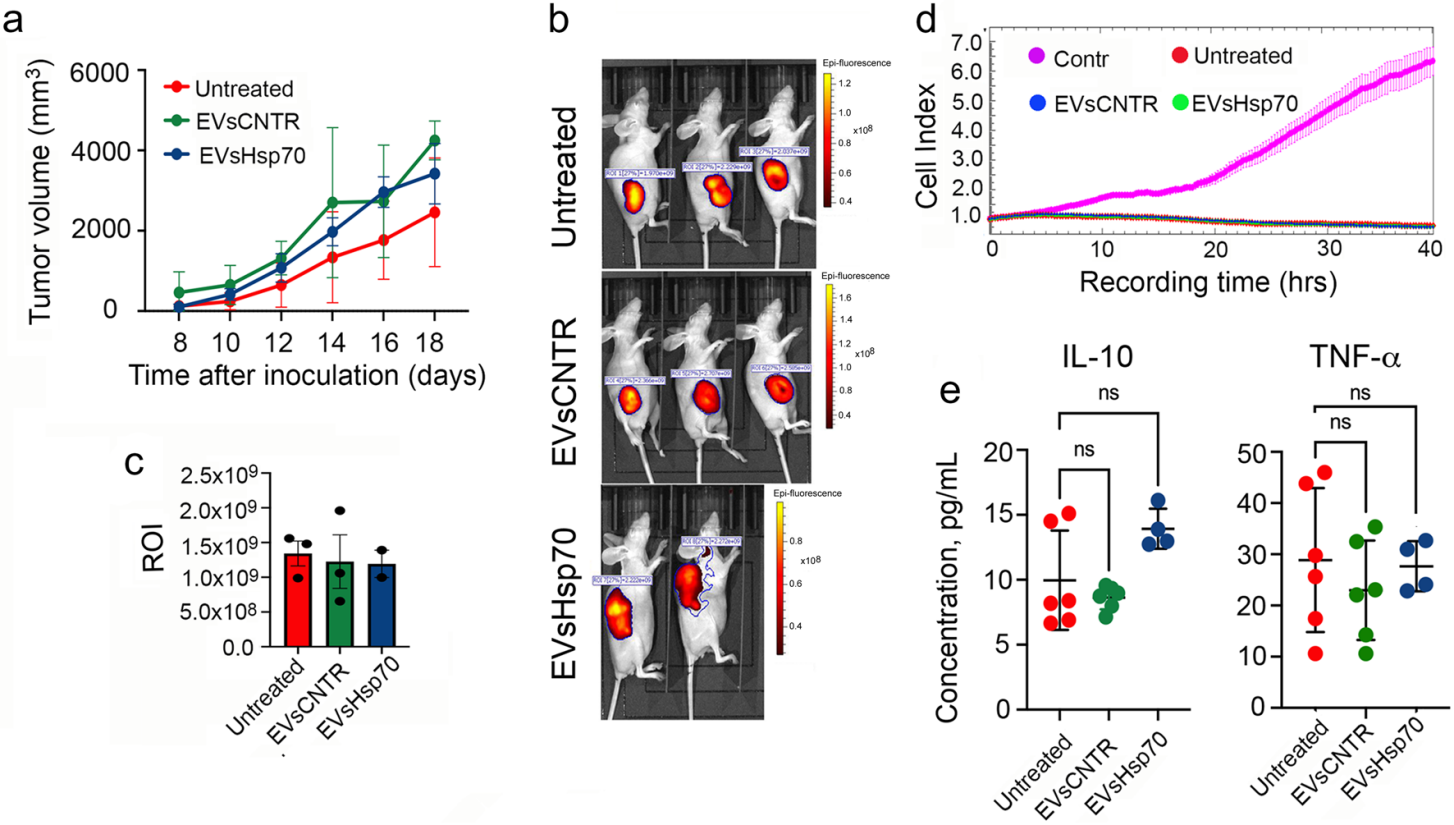
Lack of CD8 + T-cell response in Balb/c nude mice abandons antitumor effect of EVsHsp70. (**a**) Tumor volume was measured each 2nd day, starting from day 7 in Balb/c nude mice (n = 3 in each group). (**b**) tumors were monitored using the IVIS Spectrum imaging system (filters: excitation: 675 nm, emission: 720 nm). Representative biofluorescence images. (**c**) Fluorescence count of tumor lesions. (**d**) Lymphocytes from three groups for CT-26-bearing Balb/c nude mice of three experimental groups were used as effector cells in the CTL assay performed with the aid of xCELLigance (total fraction). (**e**) The concentration of two cytokines (IL-10 and TNF-α) were measured in sera of CT-26-bearing Balb/c nude mice on day 19 after tumor cell inoculation.

To prove the importance of CD8 + response in antitumor activity of EVs-Hsp70 we repeated the experiment with inoculation of CT-26 _iRFP720_ cells using Balb/c nude mice. Measurement of tumor volume each second day did not demonstrate any difference in tumor growth between mice from three experimental groups (Fig. 6a) as well as bioimaging analysis (Fig. 6b, c). The use of lymphocytes isolated from experimental animals from ‘Untreated’, ‘EVs-CNTR’ and ‘EVs-Hsp70’ groups in the CTL test led to the suppression of the proliferation of CT-26 cells, but it was exactly the same in all groups (Fig. 6d). We assume that the suppression of proliferation was associated with the activities of the NK cell. The levels of IL-10 and TNF-a also did not differ significantly in the serum of animals from all three experimental groups (Fig. 6e). Taking together, these data convince us that EVs-Hsp70 stimulate specific antitumor immunity.

### EVs with high Hsp70 content inhibit the maturation of pro-tumor macrophages

**Figure 7:**
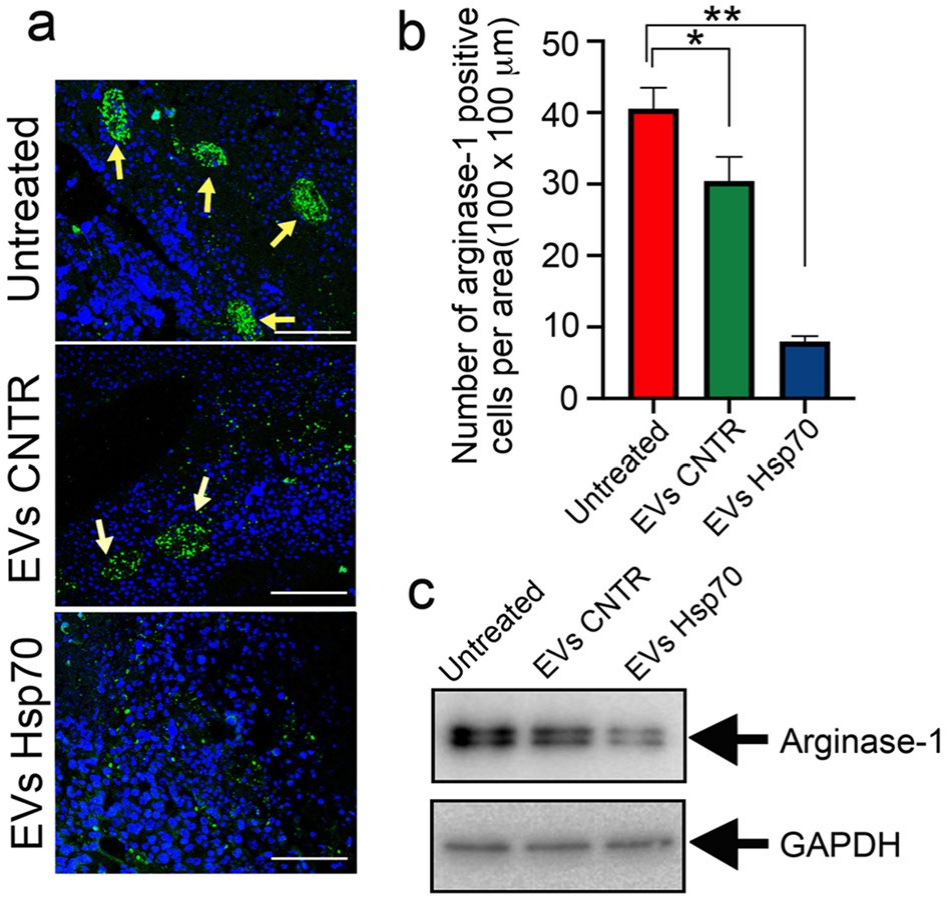
Hsp70-containing EVs inhibit the pro-tumor maturation of macrophages. (**a**) Representative histological sections obtained from B16 cell tumors on day 19 after inoculation of cells, with or without EVs, then stained with anti-arginase-1 antibody; confocal microscopy. Scale bar 100 μm. (**b**) Number of Arginase-1 positive cells as calculated per area (100 × 100 μm), 100 sections were analyzed for each treatment (*p = 0.013, **p < 0.0001, Dunnett’s multiple comparisons test). (**c**) Western blotting of B16 tumor tissue probed with anti-arginase-1 antibody. A full-length blot images used to generate the panels are shown in Suppl Fig. S10.

Recently we demonstrated that the treatment of B16 tumors with Hsp70 led to a reduction of arginase-1 positive M2 macrophages in the tumor site^28^. To assess whether EVs-Hsp70 could cause a similar effect, we prepared histological slices from tumors isolated from B16-bearing mice and probed the slices with an antibody to arginase-1. In animals from the ‘Untreated’ group, arginase-1-positive macrophages formed a high number of large solid islands of macrophages. In animals from the ‘EVs-CNTR’ group, macrophages also formed islands but less solid, whereas in tumors of the ‘EVs-Hsp70’ group, we did not observe any colonies of arginase-1-positive macrophages, although single M2 macrophages were still present (Fig. 7a). Counting the number of arginase-1-positive macrophages per area unit (100 × 100 μm) on the tumor slices demonstrated that the average number of M2 macrophages in the ‘Untreated’ group was 40.55 ± 2.93, in the group ‘EVs-CNTR it was a little lower (30.44 ± 3.38), and had dropped down to 7.98 ± 0.74 in tumors from the ‘EVs-Hsp70’ group (Fig. 7b).

The counting data was confirmed by western blotting of tumor samples from all three experimental groups, which demonstrated that the level of arginase-1 in the ‘EVs-Hsp70’ tumors was approx. eightfold lower than that in the ‘Untreated’ group (Fig. 7c).

## Discussion

The role of Hsp70-bearing EVs in tumor progression is still controversial. First, tumor-derived exosomes with membrane-bound Hsp70 were shown to increase the immunosuppressive activity of myeloid-derived suppressor cells (MDSC) via activation of STAT3^29,30^. Secondly, exosomes secreted under hypoxia with a high content of Hsp70, enhanced the invasiveness and stemness of prostate cancer cells^31^. On the contrary, the antitumor activity of Hsp70-bearing EVs from heat-treated CT-26 and B16 cells was demonstrated, and produced a strong Th1 immune response and tumor eradication in allogeneic hosts in vivo^32^. Additionally, the strong anti-tumor effects of exosomes from heated mouse colon adenocarcinoma MC38 cells was accompanied by the conversion of regulatory T cells into Th17 cells in an IL6-dependent manner^33^. Finally, Hsp70-exosomes from resistant to chemotherapy HepG2 cells showed immunoregulatory properties by stimulating an NK-antitumor response and by the production of granzyme B^34^.

Our previous data demonstrated that high antitumor immune responses could be caused by rHsp70, due to its ability to extrude its cellular homologue from a cell^16,18,35^. Suggesting that the chaperone released from cancer cells after the treatment of rHsp70 could occur in the form of EVs, we traced the paths of exoHsp70. The protein was found to penetrate tumor cells using an endocytosis mechanism and a considerable number of molecules were released to the intracellular environment within circulating Rab4 and Rab11 endosomes. The other part of early endosomes kept maturating and we found exoHsp70 in Rab5 and late Rab7 endosomes. One can suggest that at this stage, the endosome cargo can be released to the cytoplasm due to local acidification^36^ and can then enter microvesicles of multivesicular bodies (future exosomes) via the invagination of late endosomal membranes; this can explain the strong elevation of biotinylated (exo) Hsp70 in EVs found on western blots. Endogenous, unlabeled Hsp70 most probably follows the same path. Thus, we showed that Hsp70 is released from B16 melanoma cells in two distinct forms, soluble and linked to EVs. Soluble Hsp70 was considered as a carrier for immunogenic peptides, including those belonging to tumor antigens^37^. Interestingly, our methods to push out Hsp70 from living cells is similar to that occurring in heat stressed cells^33^ and results in the efficient release of EV-bound and immuno-active chaperones.

We found that EVs could penetrate B16 cells and caused Hsp70 exposition on the plasma membrane, consequently sensitizing tumor cells to NK cells. Interestingly, the concentration of Hsp70 in EVs-Hsp70 was 1000-fold lower than that of rHsp70, but the effects in the CTL test were similar. Moreover when we equalized the quantity of Hsp70 in the soluble fraction and that in EVs, it turned out that EVs-Hsp70 sensitize B16 melanoma cells to NK cells more effectively than the soluble Hsp70. This result fits well with data from De Maio group, demonstrating that extracellular membranes containing Hsp70 were at least 260-fold more effective than the free recombinant protein in the induction of TNF-α production^38^.

The results obtained in vitro were backed up in vivo by experiments with B16 melanoma and CT-26 colon carcinoma tumors. First, we proved that the addition of Hsp70-bearing EVs to tumor cells reduced tumor growth, increased animal survival rate and activated the cytotoxic T cell response, resembling results obtained with exosomes from heat-shocked tumor cells^32,33^. The data obtained on Balb/c nude mice confirmed that Hsp70 being a part of the EVs initiates particularly a specific antitumor immune response. Thus, the antitumor activity of exogenously delivered Hsp70, at least partially, may be stimulated by its intracellular conversion and release within vesicles. Secondly, the immunomodulatory activity of Hsp70-containing EVs was proved by profiling three major cytokines in the blood of tumor bearing animals; the levels of pro-cancer IL-10 was tenfold reduced, whereas anti-tumor TNF-α and IFN-γ were up-regulated to an extent dependent on the Hsp70 content in both tumor models. A high level of IL-10 is usually associated with poor prognosis for patients with pancreatic cancer^39^, melanoma and other types of cancer^40–42^. At high concentrations, TNF-α correlates with tumor regression^43^; the increase in the cytokine level associated with the application of EVs-Hsp70 argues for their significance in the mobilization of an anti-cancer immune response. A similar conclusion can be made for IFN-γ, which was shown to promote the host immune response to a variety of tumor types^44,45^. Furthermore, we analyzed an important component of the tumor microenvironment: the migration of tumor-associated macrophages (TAM) to tumor lesions and the release of factors promoting angiogenesis and metastasis^46,47^. TAMs were shown to produce a significant amount of IL-10^48^. In our experiments, tumor cells inoculated with EVs-Hsp70 demonstrated significantly lower numbers of arginase-1-positive TAMs, that correlates with low concentrations of serum IL-10. Recently we reported that recombinant Hsp70 delivered to tumor loci using hydrogel applications also delayed tumor growth and reduced the number of arginase-1-positive TAMs with a similar efficacy^28^, but the quantity of Hsp70 delivered with the EVs was 1000-fold lower than with the Hsp70-hydrogel.

Thus in both mouse cancer models, the introduction of cells together with appropriate amounts of EVs with a high Hsp70 content caused a dramatic reduction of tumor growth attributes, which was unexpected because (1) the amount of EVs was comparable with that possibly generated by the same quantity of cells and (2) the EVs did not affect tumor cell growth in vitro. We conclude that Hsp70-containing EVs affect the cells within the tumor microenvironment rather than tumor itself. Future work should elucidate the mechanisms of Hsp70-EVs participation in cross-talk between tumor cells and adjacent stromal cells.

## Conclusions

Extracellular Hsp70 occurring in soluble and embedded in extracellular vesicles (EV) forms is able to activate cytotoxicity of natural killer cells in vitro. The treatment of B16 melanoma and CT-26 colon carcinoma cells with Hsp70-containing EVs causes full-scale antitumor effect, reduction of tumor growth and prolongation of life-span that may be linked to a strong activation of innate immunity, elevation of anti-cancer cytokine levels and increase in CD8-positive cells and reduction of M2 type pro-tumor macrophages.

## Supplementary Information

Supplementary Information.

## Data Availability

All data generated or analyzed during this study are included in this published article and its additional information files.

## Abbreviations

CTL: Cytotoxic T lymphocyte assay
ELISA: Enzyme-Linked Immunosorbent Assay
EVs: Extracellular vesicles
FBS: Fetal bovine serum
IFN-γ: Interferon-gamma
IL6: Interleukin 6
IL-10: Interleukin-10,
GAPDH: Glyceraldehyde 3-phosphate-dehydrogenase
HSPA1A: Is a protein that is encoded by the *HSPA1A* gene, according to modern nomenclature, the same as Hsp70
Hsp70: Heat shock protein 70 kDA, inducible member of Hsp70 family
MDSC: Myeloid-derived suppressor cells
NK: Natural Killers
NTA: Nanoparticle Tracking Analysis
PBS: Phosphate buffered saline
PEI: Polyethyleneimine
PVDF: Polyvinylidenedifluoride membrane
Rab4, Rab5, Rab7 and Rab11: Rab GTFase 4, 5, 7 and 11
SEM: Standard error of the mean
TAM: Tumor associated macrophage
Th17: T-helper 17 lymphocytes
TNF-α: Tumor necrosis factor alfa
