# Supplementary material for: Hsp70-containing extracellular vesicles are capable of activating of adaptive immunity in models of mouse melanoma and colon carcinoma
Source: Sci Rep. 2021 Oct 29;11:21314. doi: 10.1038/s41598-021-00734-4 (PMC8556270; doi:10.1038/s41598-021-00734-4)
Supplement: Supplementary file 1 — Supplementary Information. [file 41598_2021_734_MOESM1_ESM.pdf]

Supplementary Materials

Hsp70-containing extracellular vesicles are capable of activating of adaptive immunity in models of mouse melanoma and colon carcinoma

Elena Y.Komarova, Roman V.Suezov, Alina D.Nikotina, Nikolay D. Aksenov, Luiza A.Garaeva, Tatiana A.Shtam, Alexander V.Zhakhov, Marina G.Martynova, Olga A. Bystrova, Maria S. Istomina, Alexander M. Ischenko, Boris A.Margulis, Irina V.Guzhova

Figure S1

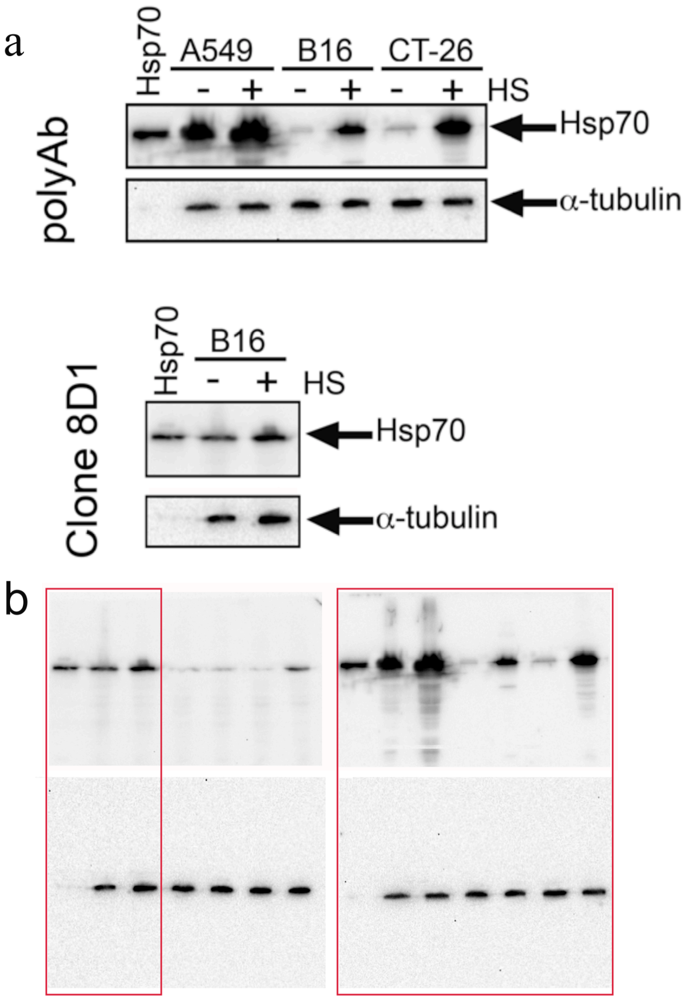

Figure S1. **Western blot demonstrating specificity of home-made anti-Hsp70 antibody to Hsp70.**

Human lung carcinoma A549 cells, mouse melanoma B16 and colon cancer carcinoma CT-26 cells were heat shocked (43°C, 6 hrs of recovery) and then subjected to western

blotting with polyclonal anti-Hsp70 (upper panel) antibody the same that were used for Hsp70-ELISA in the manuscript. B16 lysates were also used for western blotting with monoclonal anti-Hsp70 antibody (Clone 8D1) the same that was used for detection of Hsp70 in B16 EVs.

Figure S2.

Introduction: To check which transport mechanism is employed by the exogenous chaperone in B16 cells we introduced Hsp70 labeled with Alexa-488 in concentration 50 µg/ml and analysed cells using flow cytometry assay. Exo Hsp70 incorporated into 100% of B16 cells already 15 min after administration and its amount inside cells grew with time reaching maximum after 6 hrs of incubation (Fig. S2a).

To explore the presumable mechanism of intracellular transport of exoHsp70 we employed the variety of inhibitors of intracellular transport and found that clathrin-dependent endocytosis (dynasore and chlorpromazine) most probably was used to , suppress caveolin-dependent endocytosis (filipin) or to destroy lipid rafts as well as to suppress caveolin-dependent endocytosis using methyl β-cyclodextrin (MβCD). Dynasore and chlorpromazine decreased exo-Hsp70 incorporation until 41.5% and 72.5% respectively whereas both filipin and MβCD did not prevent Hsp70 penetration into B16 cells (Fig.S2b). One could conclude that exo-Hsp70 uses clathrin-dependent endocytosis pathway.

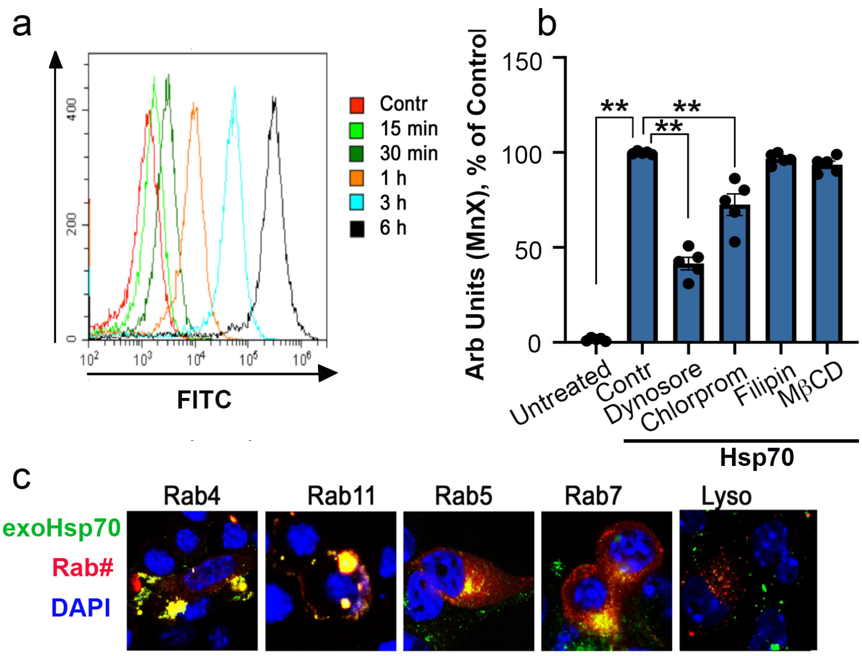

Figure S2. **Exogenous Hsp70 penetrates tumor cells using predominantly endocytosis**

(a) Flow cytometry experiment data demonstrating that rHsp70 labelled with Alexa488 accumulates inside tumor cells in time; (b) B16 cells were incubated with rHsp70 labeled with Alexa488 in presence of various intracellular transport inhibitors and then the cells were analyzed using a flow cytometry. Data of four independent experiments summarized. (c) B16 cells were transiently transfected with *rab4-*, *rab5-*, *rab7-* and *rab11- rfp* plasmids (red) and then incubated with rHsp70 labelled with Alexa488 (green) for 6 hours. Lysosomes were detected with the aid of LysoTracker (red), nuclei stained with DAPI.

To confirm that we traced the way of exo-Hsp70 in vesicular structures of B16 cells and added chaperone labeled with Alexa-488 to cells previously transfected with plasmids bearing Rab4, Rab11, Rab5 or Rab7 for 6 hrs. Non-transfected cells were also stained with LysoTracker®. Cells were studied with the aid of confocal microscopy. The highest amount of exo-Hsp70 was detected in early Rab4 and Rab11 endosomes which usually are recycled Fig.S2c). Remarkable amount of chaperone molecules was noticed in non-recycled early Rab5 and late Rab7 endosomes and only a little part of exo-Hsp70 was found to be co-localized with lysosomes.

Methods

To trace the way of exo-Hsp70 in B16 cells we first transfected cells with mApple-Rab4a-7 (for very early endosomes), or with DsRed-rab11WT (for recirculated vesicles), or with pUSP dsRed Rab5 DN (for early endosomes) or with DsRed-rab7 WT (for late endosomes) plasmids (all were purchased in Addgene, USA). Transiently transfected B16 cells settled on poly-L-lysine-coated glass slides were incubated with Hsp70 labeled with Alexa-488 (50 μg/ml) for 6 hrs, washed three times with PBS and fixed with 4% paraformaldehyde. Nuclei were stained with 4′,6-diamidino-2-phenolindole dihydrochloride (DAPI).

To reveal endo-Hsp70 on a surface of B16 cells, cells were incubated with Hsp70 labeled with Alexa-555 for 6 hours, washed three times with ice-cold PBS, stained with the cmHsp70.1-FITC antibody (kindly provided by Professor G. Multhoff, Technical University of Munchen, Germany), and fixed with 4% paraformaldehyde. Nuclei were stained with DAPI.

Fluorescence images were captured using a Leica TCS SP2 confocal microscope (Leica, Germany). To avoid possible cross-interference of various fluorochromes, images for DAPI, Alexa-555, and FITC were acquired using the sequential image recording method.

Table S1. **Nanoparticle tracking analysis (NTA) of EVs isolated from conditioned culture medium**. NTA of particle mode size and concentration. Each sample was measured in triplicate. Standard deviation (SD) is shown in parentheses.

**Vesicle source**	**Mode size (SD), nm**	**Concentration (SD), x10^{12} particles/mL**
B16 cells	89.0 (7.0)	2.8 (0.4)
B16 cells incubated with recombinant Hsp70	80.0 (4.0)	3.2 (0.4)
CT-26 cells	81.0 (11.0)	0.5 (0.07)
CT-26 cells incubated with recombinant Hsp70	89.0 (10.0)	0.7 (0.07)

Nanoparticle Tracking Analysis (NTA)

The size of EVs and their concentration were determined by NTA using the NTA NanoSight® LM10 (Malvern Instruments) analyzer, equipped with a blue laser (45 mW at 488 nm) and a C11440-5B camera (Hamamatsu Photonics K.K., Japan). Recording and data analysis were performed using the NTA software 2.3. To optimize the measurement mode, the samples of isolated vesicles were diluted 1:100, 1:1000, or 1:10,000 by PBS. In the selected dilution, each sample was measured in triplicate. The following parameters were evaluated during the analysis of recordings monitored for 60 s: the average hydrodynamic diameter, the mode of distribution, the standard deviation, and the concentration of vesicles in the suspension.

Figure S3

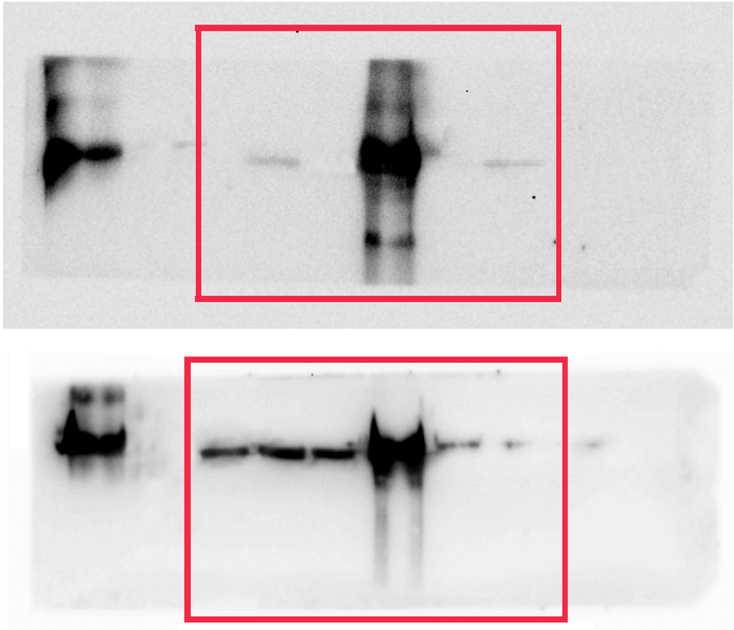

Figure S3. The full-length blot images used to generate the panels on Fig.1e in the main text

Figure S4

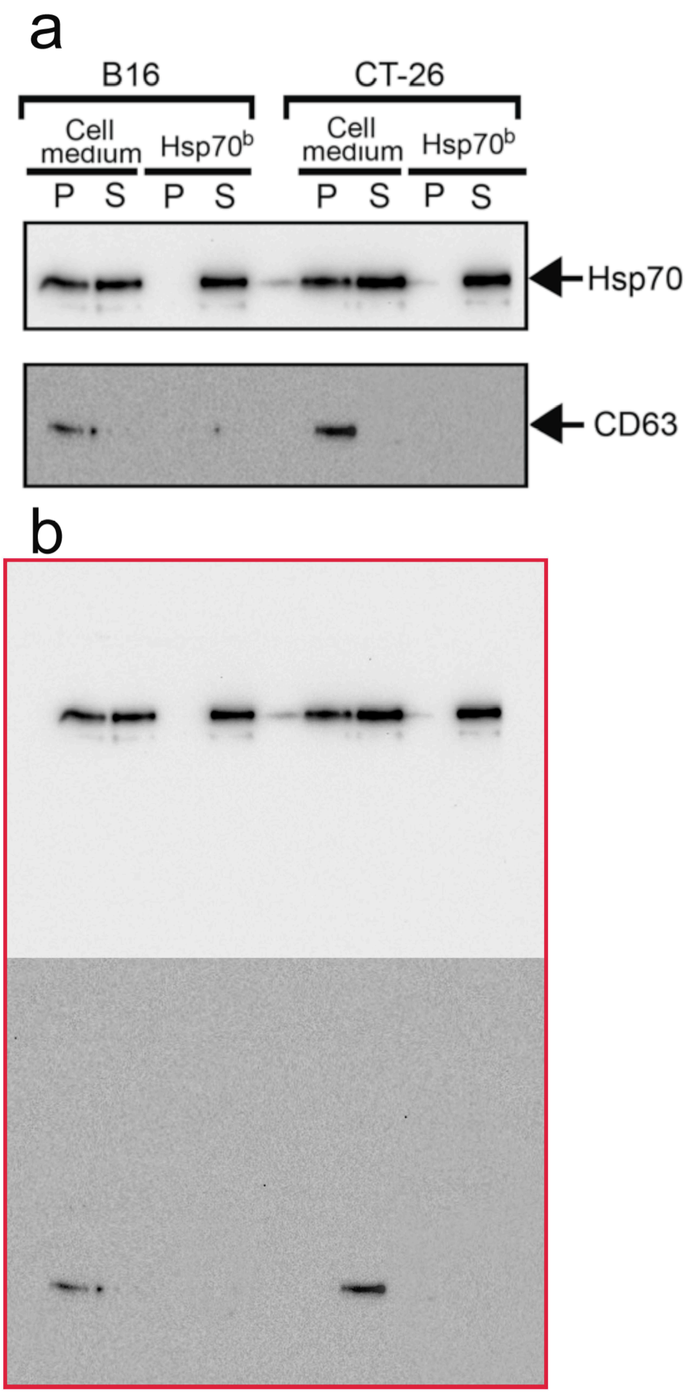

Figure S4. **There is no cross-contamination between EVs and soluble Hsp70 fractions.** To be convinced that in result of using ultracentrifugation technique for the EVs isolation soluble Hsp70 does not contaminate EVs fraction in sample 'EVs-Hsp70" and the soluble Hsp70 fraction does not contained not sedimented EVs we performed the following experiment: we have collected conditional medium from B16 and CT-26 cells incubated with eHsp70 (30 mL from two 75 cm2 flasks for each cell line) as described in Material and Methods in main text. As control we used the clean culture medium (without incubation with any cells) with added biotinilated eHsp70 (50 mg/mL). The

cell medium and Hsp70 were subjected to EVs purification protocol: first centrifuged at 400 g for 5 min, then at 20000 g for 30 min to remove big vesicles and finally ultracentrifuged at 110000 g for 2 h. As a result, we have collected Pellet (P) fractions and Supernatant (S) fractions. To get S fraction we used 10 mL of conditional medium from B16 and CT-26 cells and concentrated with aid of Amicon® Ultra-15 Centrifugal Filters Ultracel-100K (UFC910024, Merck, Germany) to final volume equal to 10μl. S fraction from "clean" medium was concentrated from 0.5 mL and then dissolved in 500 ml of Sample buffer. Pellets were dissolved in 10 μl of Sample buffer. Fractions (10 μl) were subjected to western blotting with antibody to Hsp70 and CD63. As one can see Hsp70 is not able to sedimentate during EVs isolation procedures as well as soluble fraction does not contain CD63 positive EVs (a). (b) The full-length blot images used to generate the panels on Fig.S4

Figure S5

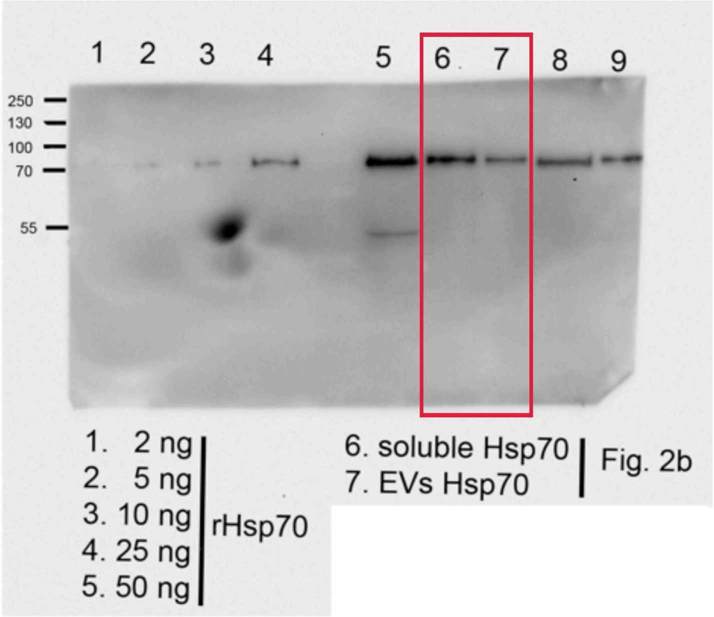

Figure S5. The full-length blot image used to generate the panel on Fig.2b in main text.

Figure S6

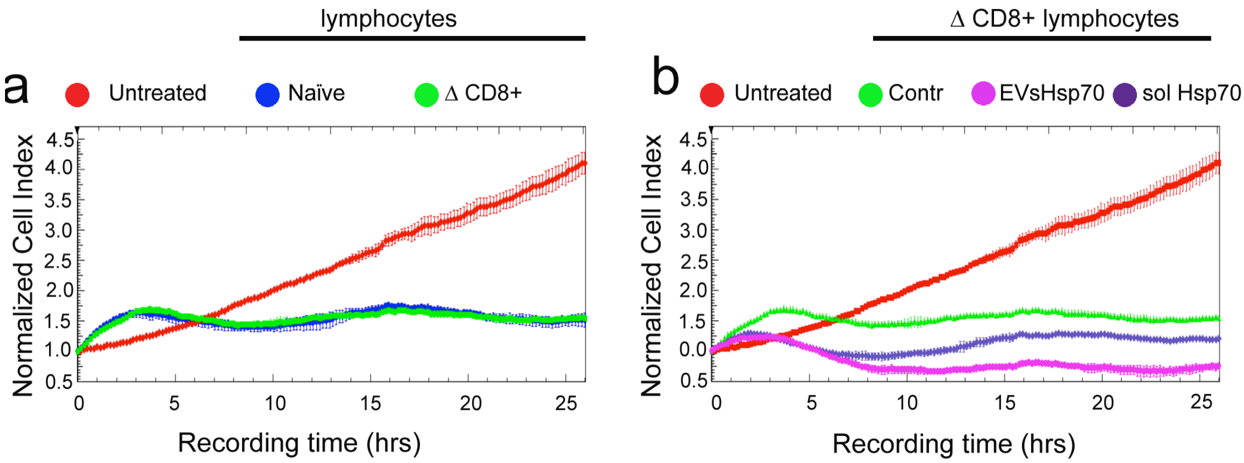

Figure S6. EVs Hsp70 sensitize B16 melanoma cells to Natural Killer cells more effectively than the same amount of soluble Hsp70. (a) CD8+ lymphocytes from naïve were removed from total lymphocytes population from C3HA mice and remained population was used as effector cells in ratio 1:100 as well as naïve C3HA lymphocytes. Data prove that CD8+ lymphocytes from naïve mice do not contribute to lymphocyte toxicity to B16 cells. (b) B16 cells were incubated with equal amount (25 ng) of soluble Hsp70 and Hsp70 connected with EVs and 6 hours later ΔCD8+ lymphocytes were added to B16 cells. Recording started when lymphocytes were added and lasted 25 hrs.

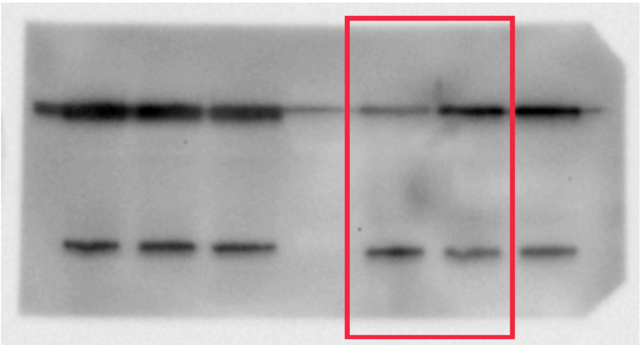

Figure S7. The full-length blot image used to generate the panel on Fig.3a in main text

Figure S8

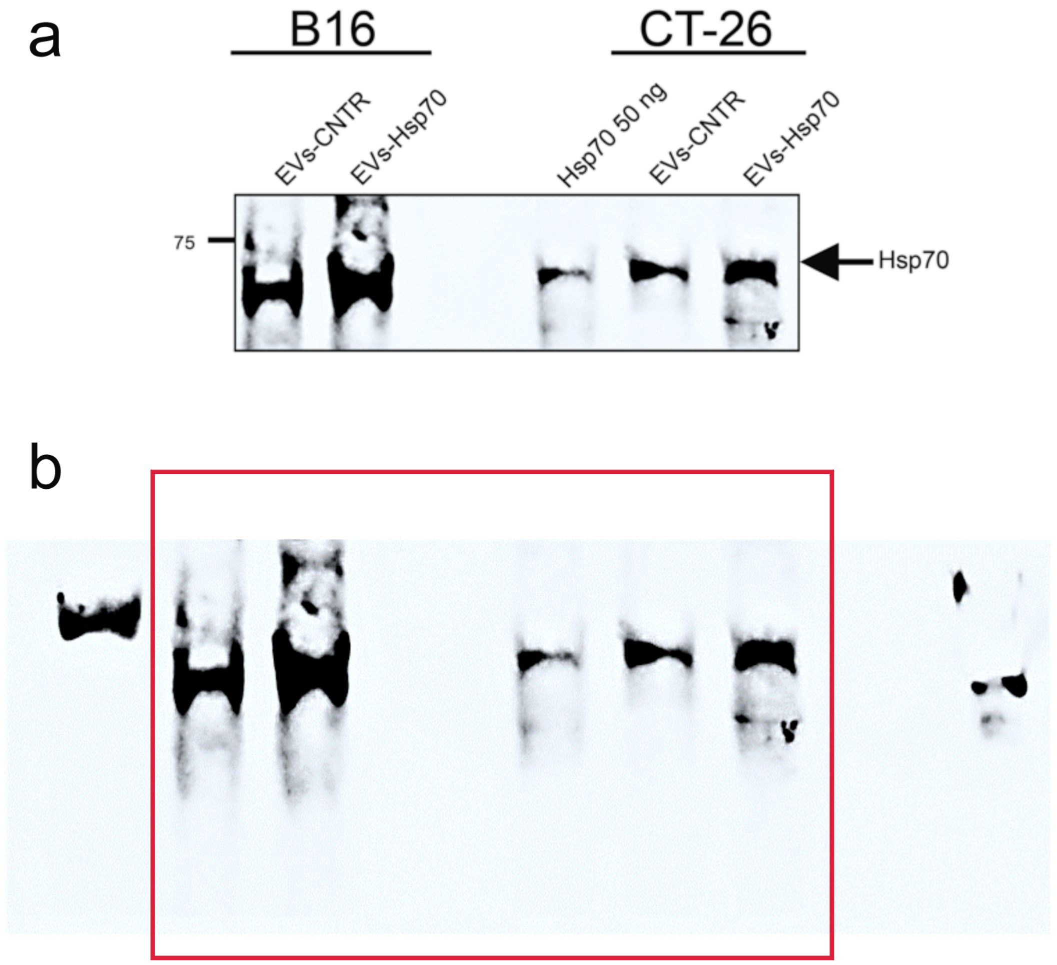

Figure S8. **Western blot of EVs released from untreated B16 and CT-26 cells and the same cells incubated with rHsp70.** EVs concentration was evaluated with aid of NTA and $8x10^9$ EVs for B16 and $2x10^9$ EVs for CT-26 was used for assay (a) .

Figure S9

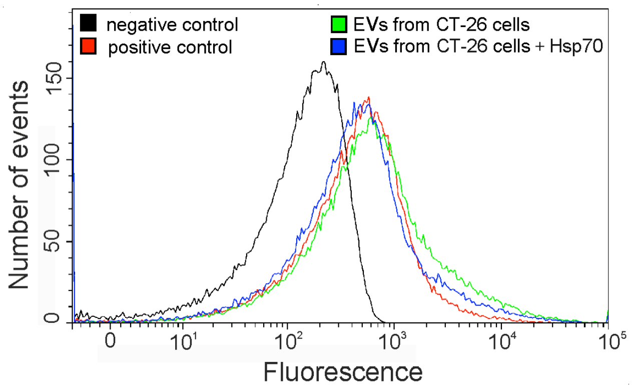

Figure S9. **EVs isolated from cell media of untreated CT-26 cell and CT-26 cells treated with Hsp70 were analyzed with flow cytometry using an Exo-FACS ready-to-use kit for analysis of CD9 positive EVs.**

Figure S10

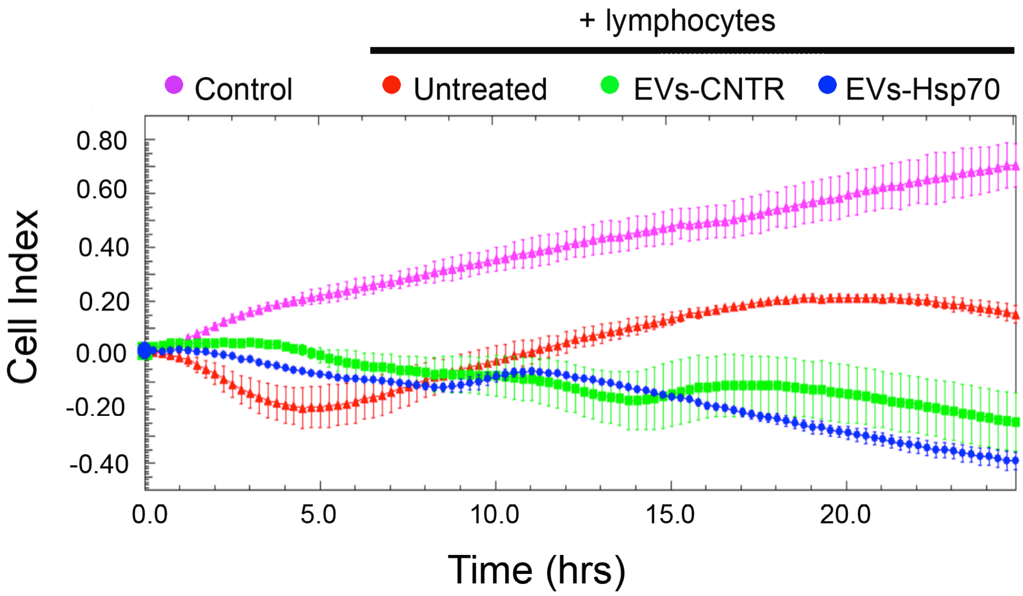

Figure S10. **CTL assay of CT-26 cells in presence of EVs-CNTR and EVs-Hsp70**. As effector cells splenocytes isolated from spleen of naïve C3HA mice. The CTL assay was performed with the aid of xCELLigence technique, which allows to trace the cell populations' behavior in real time. Importantly, lymphocytes did not cause any changes in Cell Index, so a signal change related only to adherent tumor cells.

Figure S11

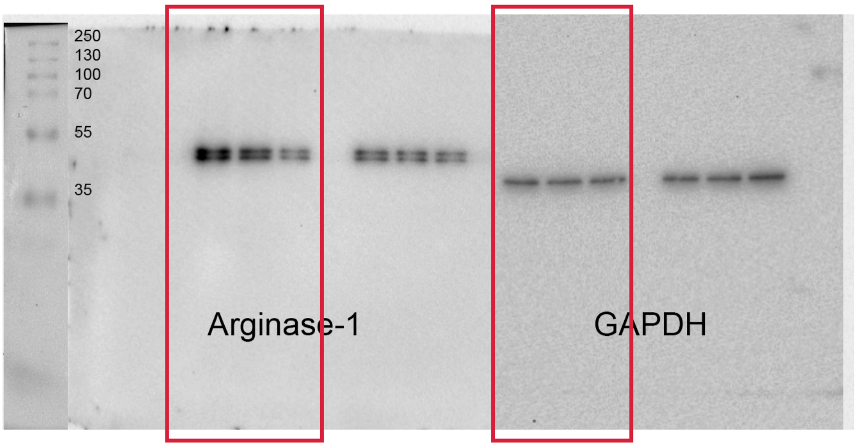

Figure S11. The full-length blot image used to generate the panel on Fig.7c in main text
